# Behavioral Variability of Endangered Northern Pig‐Tailed Macaques in a Degraded Mosaic Habitat in Northeast Bangladesh

**DOI:** 10.1002/ece3.73635

**Published:** 2026-05-25

**Authors:** Habibon Naher, Tania Akhter, Asad Ullah, Md. Shahriar Siam, Shawkat Imam Khan, Muhammad Saiful Islam, Miriam Simon, Nadine Ruppert

**Affiliations:** ^1^ Department of Zoology Jagannath University Dhaka Bangladesh; ^2^ Department of Anthropology Texas State University San Marcos Texas USA; ^3^ Department of Biology Rajshahi Cadet College Rajshahi Bangladesh; ^4^ Bangladesh National Museum Dhaka Bangladesh; ^5^ Behavioural Ecology Research Group, Institute of Biology, University of Leipzig Leipzig Germany; ^6^ Helmholtz‐Centre for Environmental Research GmbH (UFZ), Department of Molecular Toxicology Leipzig Germany; ^7^ German Centre for Integrative Biodiversity Research (iDiv) Halle‐Jena‐Leipzig Leipzig Germany; ^8^ Southeast Asia Biodiversity Research Institute Chinese Academy of Sciences Menglun Yunnan China; ^9^ Center for Integrative Conservation, Xishuangbanna Tropical Botanical Garden Chinese Academy of Sciences Menglun Yunnan China; ^10^ Yunnan Provincial Tropical Rainforest and Asian Elephant Conservation Innovation Team Menglun Yunnan China

**Keywords:** adaptation, behavioral plasticity, *Macaca leonina*, plantation use, primates

## Abstract

As wildlife habitat degradation accelerates globally, this study evaluated how habitat quality and structural complexity influence activity budgets and vertical space use in endangered northern pig‐tailed macaques *Macaca leonina* ). To examine behavioral adjustments to human‐altered environments, we studied activity and stratum use in relation to habitat type, sex–age class, and season in two wild groups: one in a multi‐crop plantation without forest access (PH) and one in a mixed natural forest–plantation habitat (MH) in and around the Satchari National Park, Bangladesh, across three seasons (August 2020–July 2021), yielding 14,591 behavioral data points. Activity budgets differed significantly between groups (*χ* ^2^: *p* < 0.001). Differences were pronounced for feeding (PH = 24.3%, MH = 21.5%; *p*_adj = 0.003), foraging (6.5%, 3.2%; *p*_adj < 0.001), and playing (1.0%, 2.0%; *p*_adj < 0.001), with trends for locomotion (42.1%, 44.2%; *p*_adj = 0.053), positioning (18.8%, 20.7%; *p*_adj = 0.053), and grooming (5.2%, 5.8%; *p*_adj = 0.091). Plant dietary diversity did not differ significantly between habitats, but plantation macaques consumed proportionally more invertebrates. Multinomial regression showed that habitat significantly influenced probabilities (*p* < 0.05) of food‐related and social activities across sex–age classes. Feeding and foraging were more likely in the plantation, whereas play was reduced. Feeding in the plantation declined significantly in summer, and adult males there were less likely to play and groom than adult females in the plantation or adult males in the mixed habitat. Vertical stratum use varied by habitat, age–sex class, and season (*p* < 0.05). Macaques spent nearly half of the day between 10 and 25 m, but plantation macaques used the ground more frequently. These findings indicate behavioral flexibility, yet simplified plantations lacking dietary and structural complexity may impose ecological and social costs that could undermine long‐term population viability jeopardizing the survival of this threatened primate in degraded habitats.

## Graphical abstract

*Macaca leonina* in Bangladesh exhibit habitat‐, season‐, and sex–age–dependent behavioral variability. Food‐related activities (*p* < 0.001, multinomial regression, shown graph) were more likely in the plantation and feeding there declined in summer; adult males in the plantation were also less likely to play and groom. Vertical stratum use varied significantly by habitat and season. Although macaques adjust behavior in simplified habitats, plantation conditions alter feeding, social behavior, and space use in ways that may entail long‐term ecological and social costs jeopardizing the long‐term persistence of this threatened species.

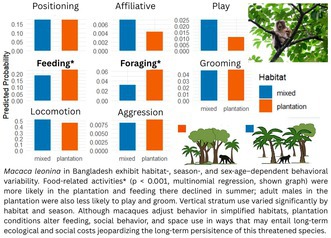

## 1 Introduction

Due to anthropogenic factors, natural habitats for wildlife worldwide, including terrestrial protected areas, are being degraded at an accelerated rate (Zhao et al. 2025). Forests are converted for agricultural use, often creating fragmented habitats and mosaics of mixed habitat patches (Haddad et al. 2015) that wild animals often adapt to by developing new behavioral strategies (Fehlmann et al. 2021). Habitat quality and resource availability fundamentally shape activity patterns of wildlife, with degraded habitats forcing increased foraging effort. For example, Barbary macaques ( *Macaca sylvanus* ) in heavily degraded forests spent significantly more time foraging and moving compared to those in more suitable habitat, while resting time dropped to its lowest levels (Ménard et al. 2013). But primates' behavioral responses to habitat alterations can vary widely according to taxon, population and the type and intensity of disturbance (e.g., Johns and Skorupa 1987; Schwitzer et al. 2011; McLennan et al. 2017). Overall evidence across multiple primate taxa consistently shows that habitat degradation increases energetic costs and alters their behaviors. Many primate taxa can persist in anthropogenically‐degraded and highly disturbed habitats (e.g., Bonilla‐Sánchez et al. 2012; Albert et al. 2014; Holzner, Rayan, et al. 2021) by changing their ranging pattern (e.g., Albert et al. 2013; Gazagne et al. 2020), activity budget (e.g., Ruppert et al. 2018), or diet (e.g., Wong and Sicotte 2007; Mekonnen et al. 2018). For example, chimpanzees show greater behavioral diversity in more variable environments, including seasonal changes (Kalan et al. 2020). Seasonality strongly shapes primates' behavioral variability. Evidence across taxa and regions highlight species‐ and habitat‐specific expressions of seasonal flexibility ranging from temperature‐driven shifts in feeding and resting in vervet monkeys (McFarland et al. 2014) to fruit‐driven changes in activity patterns in titi monkeys (Souza‐Alves et al. 2021) and great apes (Knott 2005). Behavioral variability on population and individual level is further shaped by sociodemographic factors such as group composition. Group size shows consistently strong effects on primate behavior, with larger groups traveling farther, feeding more, and exhibiting different social structures and dominance styles (e.g., Bonaventura et al. 2008; Amici et al. 2021). Sex and age effects are less studied and less consistent across various primate taxa and contexts, except where they may reflect body size differences, such as in vocal characteristics (Ey et al. 2007). In great apes, body size emerges as the most consistent driver of feeding behavior with larger males using different feeding postures and substrates than smaller females (orangutans: Cant 1987; chimpanzees: Doran 1993; gorillas: Remis 1995). Body mass scales with maximum feeding rates explaining 57%–71% of variance in primates (Nakagawa 2009) but sex‐ and age‐related effects on feeding time budgets are less consistent than body weight. For example, time spent feeding did not differ between age–sex classes in wild Japanese macaques ( *M. fuscata* ) but heavier individuals moved less and fed more on lower‐quality food compared to lighter ones (Agetsuma 2001).

Despite this high behavioral flexibility, approximately 60% of all primate taxa worldwide are now threatened, and their populations keep declining due to anthropogenic causes (Estrada et al. 2017), such as the conversion of primary forests to agricultural land, especially in South and Southeast Asia (Sodhi et al. 2010; Vijay et al. 2016). Bangladesh is a densely populated country where forests cover approximately 10% of its total land area, of which only 1.4% is legally protected (BFD 2008). Rapid human population growth and associated land‐use changes have intensified anthropogenic pressure on remaining forest habitats, leading to widespread deforestation and severe fragmentation (BFD 2008; IUCN Bangladesh 2015). Over recent decades, forest cover has continued to decline at an alarming rate due to agricultural expansion, monoculture plantations, and selective logging (Muhammed et al. 2008), further reducing habitat availability for primates and other forest‐dependent wildlife (IUCN Bangladesh 2015).

Here, the northern pig‐tailed macaque ( *Macaca leonina* ; Blyth, 1863) is one of ten native primate species (IUCN Bangladesh 2015). Its distribution ranges from north of the Brahmaputra River of eastern Bangladesh and northeastern India to southern Vietnam, Cambodia, China, Lao PDR, Myanmar, and Thailand (Roos et al. 2014). The species is found in a wide range of forest types, including tropical evergreen and semi‐evergreen, wet evergreen, moist deciduous, pine, coastal swamp, riparian, and montane forests, occurring from lowland plains to hills and mountains (roughly 50–2000 m), and persisting in secondary and degraded forests. Although mainly tree‐dwelling, *M. leonina* regularly uses terrestrial and human‐modified habitats such as forest edges, riparian corridors, agricultural areas, and degraded landscapes, especially in lowlands, where seasonal resource availability increases ground use (Boonratana et al. 2022). Across the species' range, *M. leonina* populations have been declining due to habitat loss and hunting (Boonratana et al. 2022). It is globally listed as Vulnerable on the IUCN Red List (Boonratana et al. 2022), but as Endangered in Bangladesh (IUCN Bangladesh 2015). Here, its population declined by more than 20% between 2015 and 2020 and is now restricted to a few fragmented forest patches in the northeastern and southeastern regions, occupying an area of about 4481 km^2^ (IUCN Bangladesh 2015). *Macaca leonina* is described as mainly frugivorous, semi‐terrestrial, and living in multimale‐multifemale social groups (Albert et al. 2011; José‐Domínguez et al. 2015; Gazagne et al. 2020). Group composition in Bangladesh varies, with sex ratios reported to be 1:3 (adult male to adult female; Khan and Ahsan 1981) or 1:1 (Ahmed and Naher 2021), and an adult to non‐adult ratio around 1:1.2 (Ahmed and Naher 2021). Groups are characterized by pronounced fission‐fusion dynamics and group size can vary widely with mean group sizes of 20 (Boonratana et al. 2022) to 37 individuals (Ahmed and Naher 2021) reported from Bangladesh, and up to 153 individuals (mean 141 ± SD 10) were observed in a group in a degraded habitat in Thailand (Gazagne et al. 2020). In Bangladesh, *M. leonina* does not have a pronounced breeding season but reproduces year‐round (Feeroz 2003), whereas populations in temperate North‐East India show a winter–spring breeding pattern (Choudhury 2008). At least three social groups of *M. leonina* were identified in and around the Satchari National Park in a previous study (Ahmed and Naher 2021).

Satchari National Park, located within the Raghunandan Hill Reserve Forest in the northeast of Bangladesh bordering India, is home to several endangered wildlife species that are severely exposed to anthropogenic threats (Hasan et al. 2018; Neha et al. 2020). This area comprises 112 ha of cultivated multi‐crop plantation land and 120 ha of natural forest (BFD 2016). The natural forest cover here has been decreasing due to the expansion of lemon orchards, mono‐ and polyculture plantation plots, the construction of roads, and illegal wood harvesting (Hasan et al. 2018; Ahmed and Naher 2021). Collectively, 38% of dense forests here were degraded in 1993–2006 and 42% in 2006–2019 (Masum and Hasan 2020).

Despite the conservation importance of *M. leonina* , empirical data on their behavioral ecology in human‐modified landscapes remain extremely limited, especially in Bangladesh. In particular, there is a lack of systematic, long‐term studies examining how habitat degradation through agricultural intensification influences activity budgets, vertical space use, and social behavior in this species. This knowledge gap is especially critical in Bangladesh, where rapidly shrinking and highly fragmented forests force wildlife populations to persist in agriculture‐dominated habitat mosaics with minimal structural complexity. Understanding species‐specific behavioral responses to habitat alteration is therefore essential for identifying ecological constraints, predicting population resilience, and designing targeted conservation strategies for endangered macaques in fragmented agricultural landscapes (Holzner et al. 2024).

To investigate how habitat structure influences the behavior of these macaques, we evaluated the activity budgets and stratum use of two groups in and around the Satchari National Park area. We hypothesized that activity budgets and vertical space use differ between the group that only uses the more complex mixed habitat (MH), which includes access to natural forest, and the group that lives exclusively in the plantation habitat (PH) without natural forest. We expected that food‐related behaviors such as feeding or foraging occurred more often in the food‐rich multi‐crop plantation habitat, as observed in other macaque species ( *M. nemestrina* : Ruppert et al. 2018; *M. sylvanus* : Neves et al. 2023). For grooming, mating, or other affiliative social interactions, we expected a lower occurrence in the plantation habitat compared to the more natural mixed habitat (Caselli et al. 2021; Holzner, Balasubramaniam, et al. 2021). We predicted that the activity budgets in each habitat are further shaped by season, sex, and age, with males, females, and juveniles responding differently to the ecological constraints of each habitat during different seasons. Additionally, we hypothesized that vertical stratum use, the most important spatial habitat component for arboreal species, differs habitat‐specifically in correlation with different activity types, as observed in Bale monkeys ( *Chlorocebus djamdjamensis* , Mekonnen et al. 2018), and in relation to season, sex, and age.

## 2 Methods

### 2.1 Study Area

**FIGURE 1:**
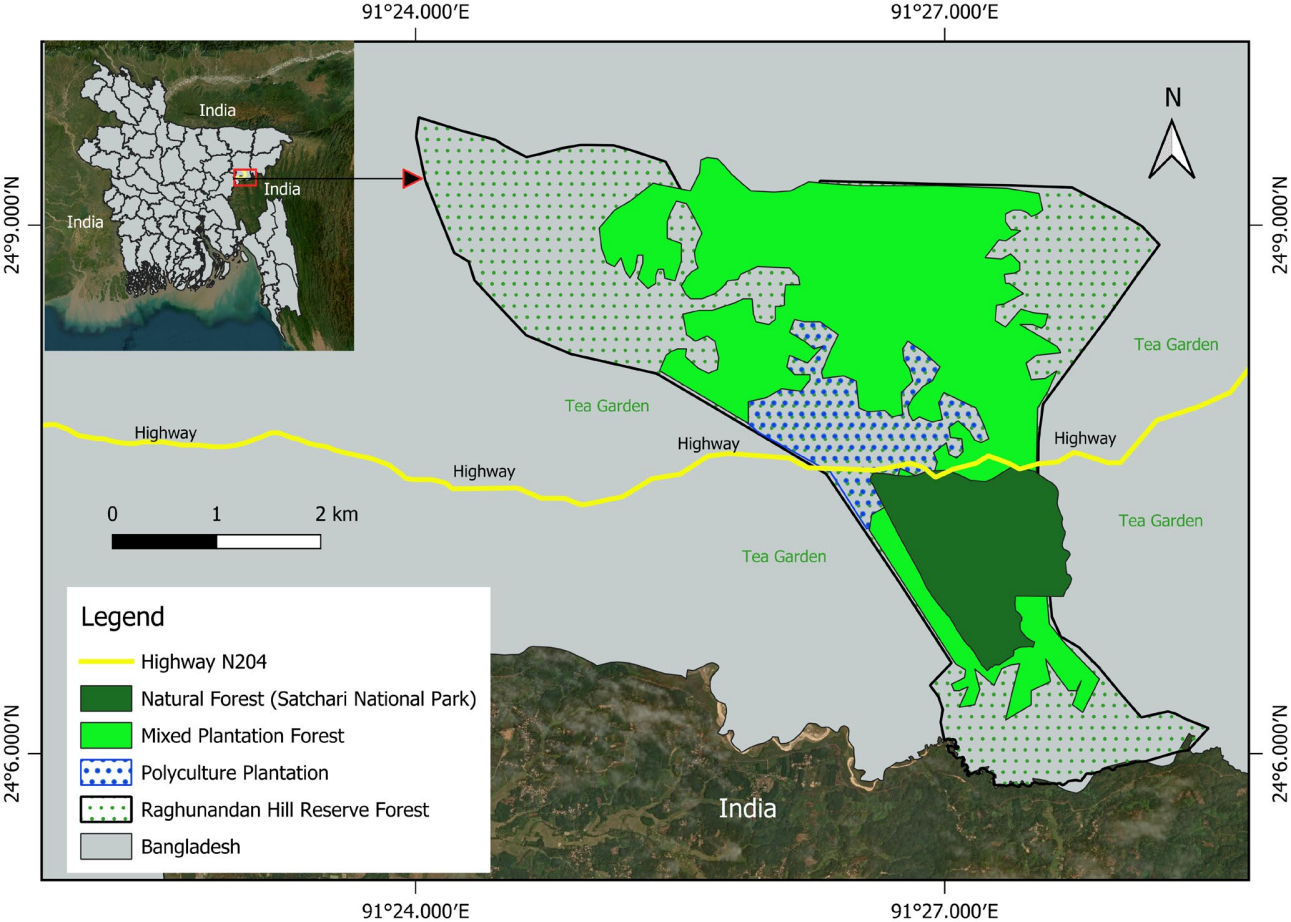
Overview map of the study area (Satchari National Park, Raghunandan Hill Reserve Forest, Habiganj District, Bangladesh: 24°07′12″ N 91°27′03″ E; also refer to Appendix 2).

Between August 2020 and July 2021, we studied two habituated *Macaca leonina* groups in and around the Satchari National Park (SNP; coordinates 24°07′12″ N 91°27′03″ E; Figure 1) including areas of polyculture plantations and natural forests. Satchari National Park is a small (232 ha), semi‐evergreen forest located in a transition zone between the Indian subcontinent and the Indo‐Chinese ecological regions (Sharma 2006). The study area's topography is composed of upper tertiary rocks dominated by sandstone and undulating slopes and hillocks with a height of 10–50 m (commonly known *astila*), stretching from south to north (Rizvi 1970). Located in the Habiganj District in northeast Bangladesh, it is part of the Raghunandan Hill Reserve Forest (BFD 2016) and one of the most biodiverse forests in Bangladesh (Masum and Hassan 2020). The national highway (N204) bisects the study area, which is further fragmented by tea ( *Camellia sinensis* ), oil palm ( *Elaeis guineensis* ), teak ( *Tectona grandis* ), and rubber ( *Hevea brasiliensis* ) monoculture plantations, multicrop agricultural fields, lemon orchards, human settlements (villages of the Tripura ethnic community), and other structural development (e.g., mosques, local forest department offices, dormitories, and rest houses) (Neha et al. 2020). Three natural ponds are located in the study area, one in the mixed plantation forest habitat and two in the polyculture plantation habitat. Seven streams, locally known as *chara*, travers the study area (Al‐Razi and Naher 2021) that experiences significant water flow during the monsoon season that can result in flash floods. The year is divided into three seasons, that is, winter (November to February), summer (March to June), and monsoon (July to October). Seasonal weather variation in different months impacts leaf and fruit production and thus, natural food availability for primates at the study site (Hasan et al. 2018; Neha et al. 2020). During the one‐year study period, we used a weather station (Bresser 7002580 5‐in‐1) to record temperature, relative humidity, and rainfall at the study site (Appendix 1).

Six sympatric primate species live in Satchari National Park: the northern pig‐tailed macaque ( *M. leonina* ), rhesus macaque ( *M. mulatta* ), western hoolock gibbon ( *Hoolock hoolock* ), Phayre's langur ( *Trachypithecus phayrei* ), capped langur ( *T. pileatus* ), and the Bengal slow loris ( *Nycticebus bengalensis* ) (Naher et al. 2022). The study area shelters three groups of *M. leonina* (Ahmed and Naher 2021) that are isolated from other populations. One group (referred to as group MH in this study) lives in a mixed habitat composed of natural forest and polyculture plantations, while the other group (PH) is restricted to a polyculture plantation including oil palm stands without access to natural forest (Appendix 2). A third group that partially overlaps with group PH (Ahmed and Naher 2021) inhabits the natural forest edge near human settlements and lemon orchards. It was not followed during this study due to a lack of manpower and time.

### 2.2 Habitat Types

**FIGURE 2:**
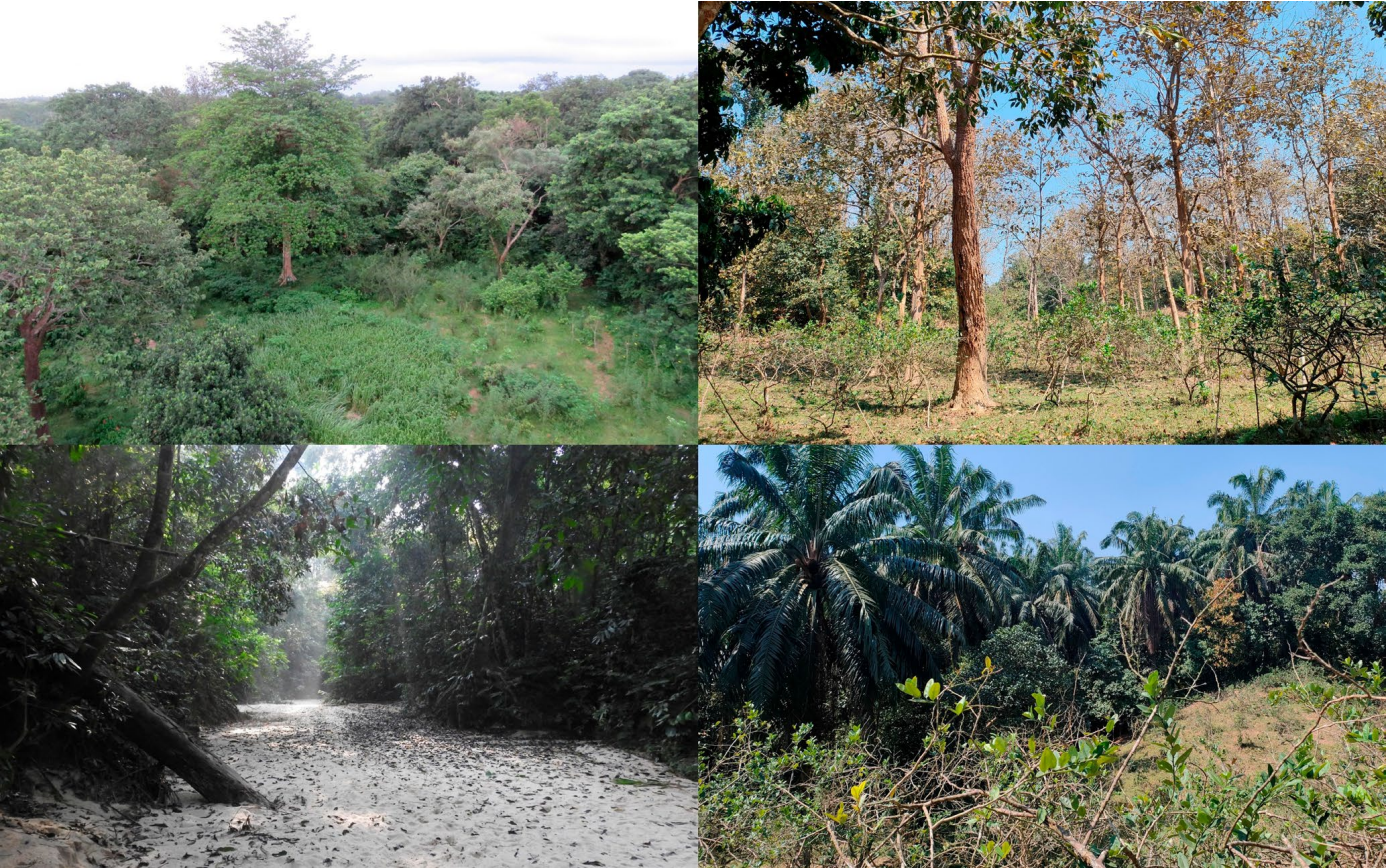
Habitat types: Left (top and bottom)—Mixed habitat with natural forest and plantation mosaics; Right—Plantation habitat with cultivated teak (*Tectonia grandis*) and lemon (*Citrus* sp.) (top), and oil palm ( *Elaeis guineensis* ) (bottom).

During the study period, we assessed the habitat structure within the ranging area of each macaque study group (Appendix 2). Within the home ranges of both groups, we established 10 m wide transects along accessible trails and arranged vegetation plots measuring 20 m × 20 m each alternately along each transect, with a spacing of about 50 m between successive plots. Within the mixed habitat site occupied by group MH, we established 43 plots (17,200 m^2^) along 18 transects (total length: 3930 m), and 73 plots (29,200 m^2^) along nine transects (total length: 5795 m) in the plantation habitat site occupied by group PH. Fewer plots were made in the mixed habitat due to its denser vegetation and less accessible topography compared to the plantation (Figure 2). We measured and identified all trees > 15 cm diameter at breast height (DBH) noting DBH and estimated tree height using a clinometer.

### 2.3 Behavioral Data Collection

During the onset of the study in July 2020, we followed the groups for 5 days to become familiar with the macaques at the site and their ranging patterns, to select the study groups, and to test research protocols to avoid observer bias. The two selected groups were already adapted to humans due to the high presence of visitors and forest‐dependent inhabitants at the study area and did not show a fleeing response when observed by the researchers. From August 2020 onwards, we followed both groups with two trained observer teams for two to five consecutive days every month from dusk until dawn. During data collection, observers always maintained a safe observation distance of at least 3 m from the macaques. Both groups were followed from the moment they left their sleeping site after sunrise (4:30–6:45) until they had settled at their sleeping site after sunset (17:00–19:30), and the locations of the sleeping sites were recorded with GPS to find the groups the next morning. Both groups ranged near the highway, but their home range cores did not overlap (Appendix 2).

Three trained observers in each team followed one group each on the same day to collect instantaneous group scan and ad libitum data using a standardized protocol (Altmann 1974) and an ethogram (Table 1) adapted from Ruppert et al. (2018). During each group scan, individual data on activity and stratum use was recorded on three adult males, three adult females and three nonadult individuals (i.e., independent juveniles whose sex could sometimes not be determined; dependent infants were not included in the data collection; Appendix 3). Individuals in sight were randomly selected while trying to avoid repeated scans of the same individuals (e.g., dominant or bold individuals that are more often near observers). The observers actively searched for individuals if not all nine targeted sex–age classes were in sight during the scan. If the nine target individuals were not detected within 10 min, observers marked the missing individual(s) as “out of sight” and stopped recording data until the next group scan. The 10‐min group scan observation period was followed by a 30‐min pause during which qualitative ad libitum observations were recorded to complement the quantitative data set. All individuals recorded within the 10 min scan time were included in the analysis (excluding the “out of sight” data points). The observers could not identify individual animals, except for a few individuals with prominent morphological features, because of the large sizes of the groups, making prolonged focal animal observations impossible at the time.

**TABLE 1:** Ethogram of *Macaca leonina* (modified from Ruppert et al. 2018).

Activities	Definition
Feeding Fi	Food ingestion by chewing and swallowing food items. The following food‐related categories were also included in Fi for the data analysis: (1) Food processing in the mouth, e.g., opening a fruit, rubbing and cleaning fruits or cracking seeds with the teeth. (2) Cheek pouch feeding: Eating food previously stored in cheek pouches. (3) Drinking: Bringing water to the mouth, swallowing water, licking water from leaves.
Foraging Fo	Searching for food, i.e., locomotion or standing while intentionally looking for food and/or handling leaves, twigs, soil and other material with the hands to obtain food items.
Locomotion L	Directed movement to another place without foraging‐related intention: Walking, running, jumping, or climbing without any other behavior.
Positioning P	Sitting, standing, or lying down without any other behavior; resting.
Grooming G	Cleaning of the body fur with hands, feet or mouth. This activity was recorded when (1) an individual was grooming itself during resting periods; (2) an individual was grooming another individual (active groomer); (3) an individual is groomed by another individual (passive groomee).
Playing AP	Social affiliative play between two or more individuals including play chase, play fight, mock bite, etc.
Affiliative Af	All positive social interactions except grooming, playing and mating, e.g., touch, friendly mount, embrace.
Mating M	Male mounting a female with intromission of penis.
Aggression Xa	All agonistic behaviors, including chase, attack, teeth display, bite.

The observers recorded stratum data of each scanned individual to examine their vertical habitat use and the level of terrestriality of both study groups. The stratum an animal occupied during a scan was visually estimated according to four pre‐defined categories, that is, F0: ground level, F1: lower stratum (above ground to approximately 10 m), F2: middle stratum (between approximately 10 and 25 m) and F3: high stratum (> 25 m in the canopy) following Ruppert et al. (2018). When an individual's activity was recorded as “feeding”, the observers trained in botanical studies noted the food item and plant taxon (identified to the lowest taxonomic level) to calculate feeding frequencies (Appendix 4) for the dietary diversity assessment.

Once per hour, the observers conducted a rapid group count by counting all visible individuals, including infants. As the groups were large and single individuals could not be identified, and to make sure that individuals were not double counted during a group count, one observer quickly scanned all visible individuals in the troop from right to left (360 degrees) in all strata during a group count scan. The precise sex–age classes of the individuals could not always be determined during these rapid scans due to obstructions by vegetation or quick movements of the animals. The maximum number of individuals in each group counted in each month was used as the best estimate to calculate the mean group sizes (Appendix 5).

To avoid observer bias, the same observer was responsible for assessing the activity of the scanned individual and the stratum category. The second observer wrote down the data and observed the time available for a group scan and pause, and the third observer helped to find and track the individuals, conducted the group count, and recorded ad libitum data. During data collection, the team members actively communicated with each other during data collection to verify observations.

### 2.4 Data Analysis

#### 2.4.1 Activity Budgets

The percentage of time (*T*_a_) spent on an activity (*a*) during the day was estimated using the formula *T*_*a*_ = (*n*_*a*_ × 100)/N where *n*_*a*_ is the number of records of activity *a* and *N* is the total number of records (Naher et al. 2022). The data set was tested for normality using the Kolmogorov–Smirnoff test. The non‐parametric *χ* ^2^ test of goodness of fit was used to compare activity budgets as well as stratum use in mixed versus plantation habitats over three seasons. Firstly, to compare activity budgets, a 2 × 2 contingency table was created, showing the counts of the focal activity and all other activities combined across the two habitat types (mixed vs. plantation). A *χ* ^2^ test was then conducted on each table to determine if the frequency of the focal activity varied significantly between habitats (Appendix 6). Secondly, to compare the activity budgets per habitat and season, we constructed contingency tables cross‐tabulating observed activity categories against habitat type (mixed vs. plantation). *χ* ^2^ tests were then performed on these tables to evaluate whether the distribution of activities differed significantly between habitats within each season (Appendix 7). To account for the multiple tests performed (one per activity/multiple comparisons per season), *p*‐values were adjusted using the false discovery rate (FDR) method to reduce the risk of false positives. All tests were performed using base functions in R (version 4.3.1).

#### 2.4.2 Multinomial Logistic Regression Models

To assess how habitat type influences macaques' behaviors, we conducted two sets of multinomial logistic regression models, as our dependent variables are nominal and have more than two unordered categories (Koster and McElreath 2017). Individuals of both groups could not be reliably identified; therefore, instantaneous group scan samples choosing nine random individuals (i.e., any visible three adult males, three adults females and three juveniles) were treated as observation‐level data.

Both models were fitted in R using the function “multinom” from the package “nnet” version 7.3.19. For the calculation of the *p* value, the *z* values were calculated, and Wald tests were performed using the function “pnorm”. The multicollinearity among the predictors was assessed using the Variance Inflation Factor (VIF) with the function “vif” (package “car” version 3.1.2; Fox and Weisberg 2019). The predictors did not show collinearity issues, ensuring the reliability of the regression model. We used a likelihood ratio test (LRT) comparing models that include interactions with models without interaction terms using the function “lrtest” (package “lmtest” version 0.9.40; Zeileis and Hothorn 2002) to ensure that the interactions included in the model significantly improved the fitness of the model. Finally, model stability was assessed by performing a nonparametric bootstrap resampling procedure with 1000 iterations with the function “boot_func” (package “boot” version 1.3‐31; Canty and Ripley 2016).

To investigate the influence of habitat on macaques' behavior in conjunction with sex–age class, we first fitted “activities” as the response variable with eight levels, excluding “mating” because too few observations were made for this activity, skewing the model. “Habitat type” (mixed or plantation), “sex” (adult male or adult female), “age” (adult or nonadult), “season” (monsoon, winter or summer) and use of “stratum” (F0, F1 or F2; excluding F3 as almost no observations were made in this stratum) were included as predictors in the model and the neutral activity of “positioning” was established as the reference response level. Further, we included three two‐way interactions: (i) “sex and habitat”, to address whether adult males and adult females adjust their behavior differently in both habitat types; (ii) “age and habitat”, because non‐adult individuals (i.e., independent juveniles) could potentially act differently than adults in either habitat type; and (iii) “stratum and habitat”, since a change in vertical space use in either habitat could influence observable activities.

Second, we fitted a model with “stratum” as a response variable with four levels to investigate the influence of habitat on the spatial behavior of macaques. Similarly to the first model, we included “habitat”, “sex”, “age”, and “season” as predictors, and the stratum level F0 was set as the reference response level. The highest level F3 was removed for model stability, as too few observations were made in this stratum. To account for interactive effects, we included three interactions in the model, (i) “sex and habitat”, (ii) “age and habitat”, and (iii) “season and habitat”.

#### 2.4.3 Dietary Diversity

Food items ingested during feeding scan observations were noted down to species level whenever possible. From these observations, percentages of ingested plants species were calculated (Appendix 4). Unidentified plants, other plant matter (e.g., bark, twigs, dry leaves, fungus, grass) as well as invertebrates (e.g., insects, spiders, ants and termites) were also noted down and percentages calculated. Plant‐specific dietary diversity was quantified separately for each macaque group using the Shannon–Wiener diversity index (*H*′), calculated as $H^{\prime }=-\sum p_{i}\ln p_{i}$, where $p_{i}$ represents the proportional feeding frequency of food item *i* relative to the total number of feeding records (Appendix 4). Feeding frequencies were derived from all observed “feeding” activity data recorded during the group scans and R function “diversity” (package “vegan” version 2.7.2) was used to calculate *H*′.

Species richness (*S*) was defined as the total number of food taxa recorded per group. Evenness was assessed using Pielou's index (*J* = *H*′ ln *S*), which quantifies the equitability of feeding records among groups. Variance estimates of Shannon diversity were calculated following Hutcheson (1970), and differences in *H*′ between groups were calculated using Hutcheson's *t*‐test, which incorporates diversity variance and unequal sampling effort into the test statistic in R. Degrees of freedom were estimated using the Welch–Satterthwaite approximation. All diversity calculations employed natural logarithms, and statistical significance was evaluated at *α* = 0.05.

### 2.5 Ethics Statement

This non‐invasive study on wild macaques was conducted with the approval from the Bangladesh Forest Department (permit number 22.01.0000.101.23.2020.1135 issued to H.N.) and adhered to the animal ethics standards of Jagannath University.

## 3 Results

### 3.1 Habitat Structure

#### 3.1.1 Mixed Habitat

In total, 637 plant individuals of 60 species in 26 families were assessed in the mixed habitat resulting in a mean stratum height of 8.7 m (2.5–26 m; median 7 m) with a mean DBH of 55.6 cm (3–300 cm; median 38 cm). Here, plant species were distributed in a scattered and irregular pattern, reflecting a heterogeneous vegetation mosaic. The five most abundant families in terms of individual tree numbers were Moraceae (24.5%), Euphorbiaceae (13.3%), Malvaceae (9.4%), Arecaceae (8.0%), and Lamiaceae (7.1%). *Aidia densiflora*, *Antidesma ghaesembilla*, *Aporosa octandra*, *Artocarpus chama*, *Elaeis guineensis* , *Elaeocarpus floribundus*, *Ficus heterophylla*, *Microcos paniculata*, *Streblus asper*, and *Tectona grandis* were the most abundant species. The relative importance of oil palm ( *E. guineensis* ) in the mixed habitat was 8.7% (calculated as the sum of relative frequency and relative dominance).

#### 3.1.2 Plantation Habitat

In total, 1914 individuals of 72 species in 34 families were identified in the plantation habitat with a mean stratum height of 7.3 m (2–30 m; median 6 m) and a mean DBH of 46.2 cm (8–274 cm; median 31 cm). The plantation habitat was dominated by Moraceae (37.5%), Rhizophoraceae (11.8%), Myrtaceae (7.1%), Euphorbiaceae (6.8%), and Arecaceae (6.5%). This habitat consisted of planted and clumped patches of *Acacia mangium* , *E. guineensis* , *Eucalyptus globulus* , *S. asper* and *T. grandis* . Other abundant species included *A. octandra* , *A. chama* , *F. hispida* , *F. heterophylla* , and *M. paniculata*. The relative importance of the oil palm here was 43.4%.

### 3.2 Macaque Group Characteristics

The observers spent a total of 689 h during 53 days in the field. Group MH was sampled on 45 days resulting in 933 group scans, and group PH on 49 days (951 group scans). A total of 14,591 individual scans (excluding 1356 “out of sight” data points) were recorded for both groups (MH: 7804; PH: 6787). Group MH consisted of a mean number of 89.9 (±35.0) individuals and group PH of 75.2 (±25.5) individuals.

### 3.3 Diurnal Activity Budgets

Overall, the macaques spent almost half (43.2%) of their daytime locomoting, followed by feeding (22.8%), and positioning (19.8%). They spent about the same amount of time foraging (4.7%) and grooming (5.5%). Other social behaviors like playing, mating, and other aggressive or affiliative interactions occurred less often (4.0%; Appendix 6). The proportion of time macaques spent on certain activities varied significantly between the two study groups (*χ* ^2^ = 124.75, df = 8, *p* < 0.001, Appendix 6). Both groups spent similar amounts of time on locomotion (means: PH = 42.1%, MH = 44.2%), positioning (18.8%, 20.7%) and grooming (5.8%, 5.2%), but the PH group spent significantly more time feeding (24.2%) and foraging (6.5%) than the MH group (21.5%, 3.2%, respectively). The MH group spent significantly more time playing (2.0%) than the PH group (1.0%). There were also significant differences in the activity budgets of both groups in the three different seasons (monsoon *χ* ^2^ = 73.7, df = 8, *p*_adj < 0.001; summer *χ* ^2^ = 41.3, df = 8, *p*_adj < 0.001; winter *χ* ^2^ = 59.4, df = 8, *p*_adj < 0.001; Appendix 7).

#### 3.3.1 Factors Influencing Activity Budgets

The multinomial regression model (Appendix 8) revealed several significant effects of habitat, sex–age class, stratum use, season, and their respective interactions on macaques' activity budgets compared to the baseline response level (positioning P).

##### 3.3.1.1 Habitat

**FIGURE 3:**
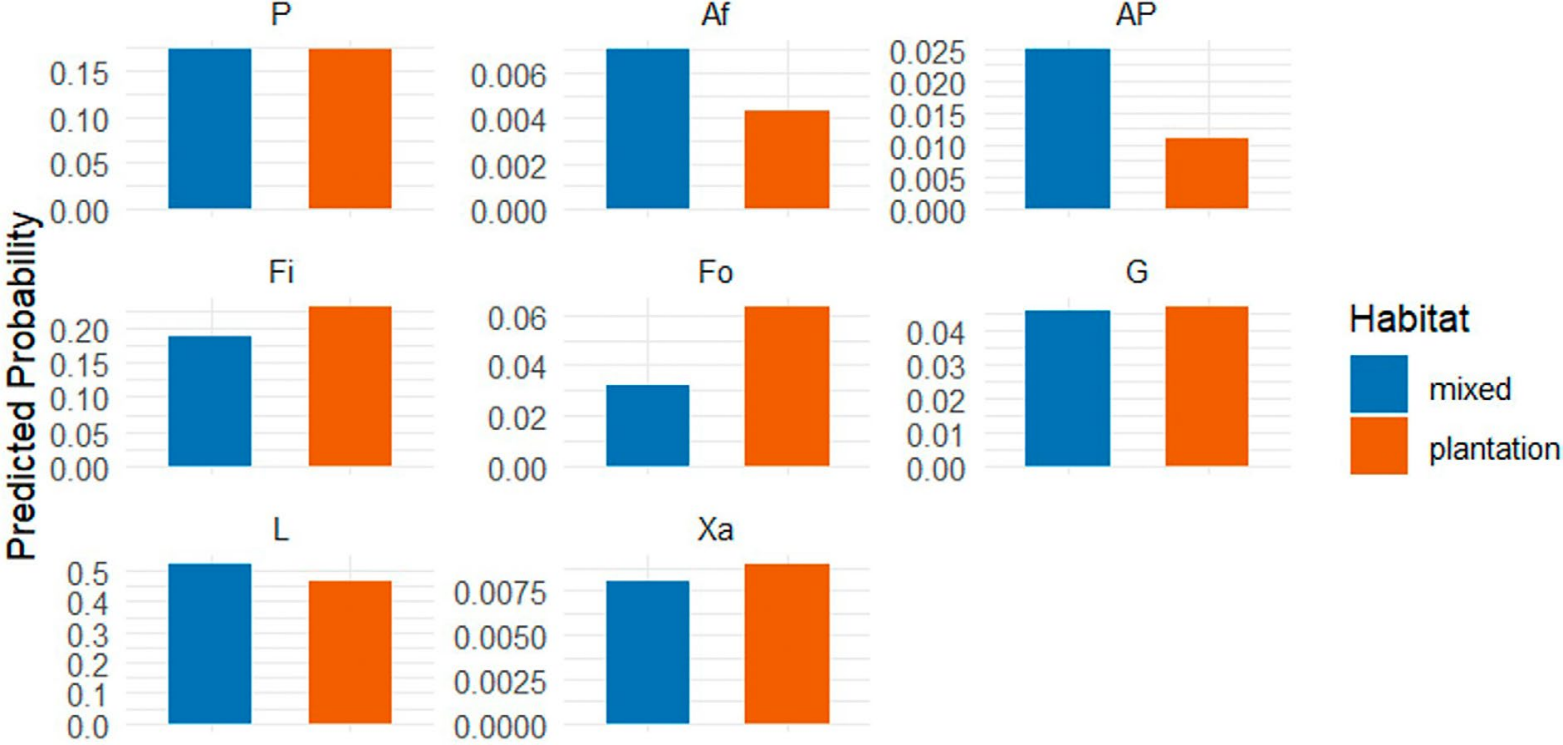
Probabilities predicted for each activity of *Macaca leonina* according to habitat type (mixed or plantation) estimated from the multinomial regression model (Appendix 8). The *y*‐axis represents the probability predicted by the model of observing a given activity, ranging from 0 to 1. Feeding (*p* < 0.001) and foraging (*p* < 0.001) showed significant differences between the two habitats, but interactions need to be further taken into account. Af, Affiliative behaviors; AP, Playing; Fi, Feeding; Fo, Foraging; G, Grooming; L, Locomotion; P, Positioning; Xa, Aggression.

The main factor “habitat” influenced the probability of feeding (Fi) and foraging (Fo) activities (both *p* < 0.001) significantly positively when averaged across all sex–age classes, showing that these food‐related activities were more likely to be observed in the plantation habitat compared to the mixed habitat (Figure 3).

##### 3.3.1.2 Sex and Age

**FIGURE 4:**
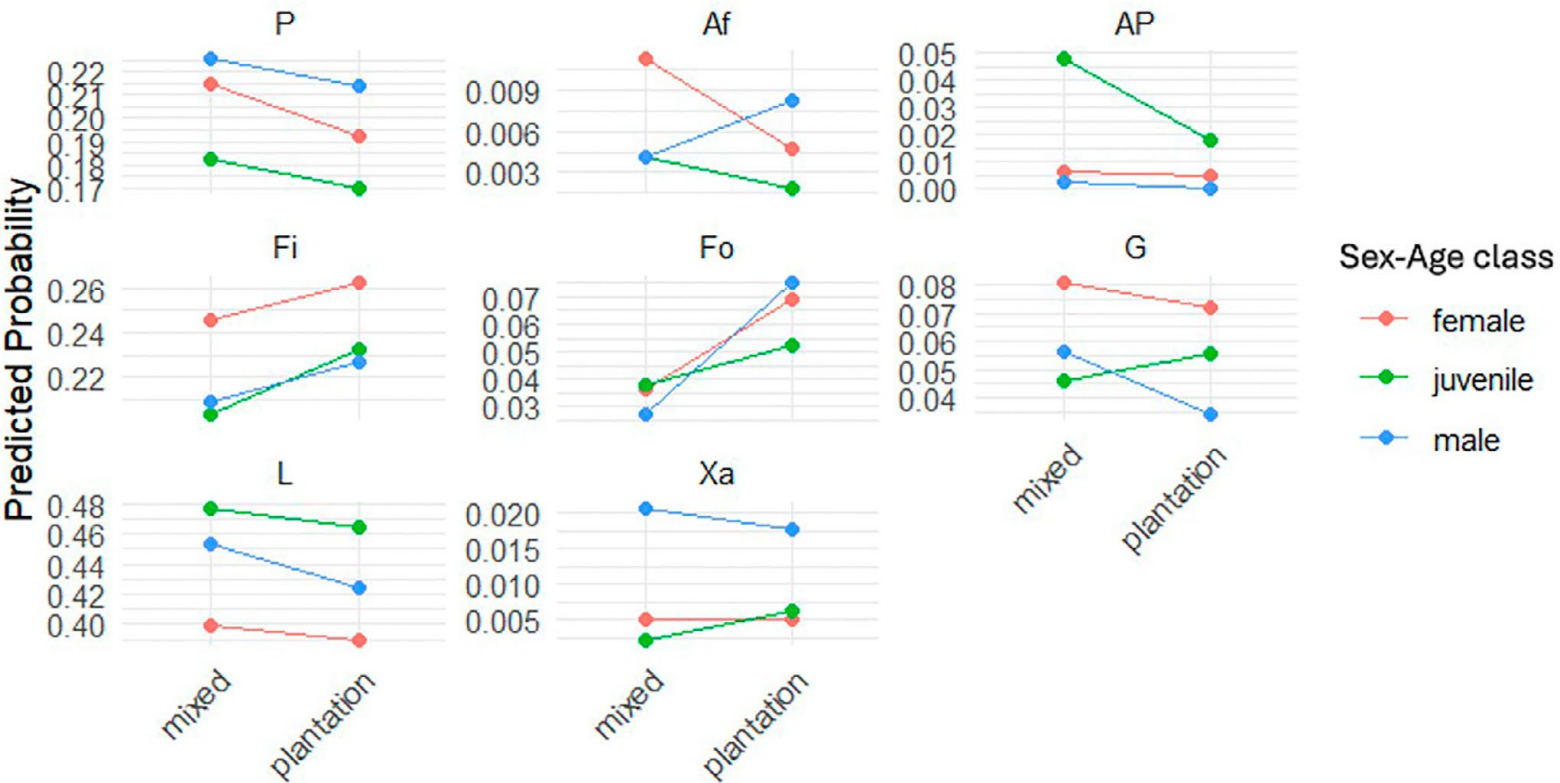
Probabilities predicted for each activity of *Macaca leonina* according to habitat type (mixed or plantation) and sex–age class (adult male, adult female, or juveniles of both sexes) as estimated from the multinomial regression model (Appendix 8). The *y*‐axis represents the probability predicted by the model of observing a given activity, ranging from 0 to 1. Affiliative behaviors (*p* < 0.001) and grooming (*p* = 0.03) were significantly influenced by habitat regarding the interaction with sex–age class. Af, Affiliative behaviors; AP, Playing; Fi, Feeding; Fo, Foraging; G, Grooming; L, Locomotion; P, Positioning; Xa, Aggression.

Playing (AP, *p* < 0.001) and grooming (G, *p* = 0.03) were both less likely to be observed in adult males in the plantation compared to adult females in the plantation or adult males in the mixed habitat. However, other affiliative behaviors (Af, *p* = 0.002) were more likely to be observed in males in the plantation habitat than in adult females in the plantation habitat or adult males in the mixed habitat. Adult males were less likely observed feeding (Fi, *p* = 0.02) or foraging (Fo, *p* = 0.04) than adult females but significantly more likely showed aggression (Xa, *p* < 0.001). Regardless of habitat, age influenced playing (AP) and locomotion (L) significantly positively (both *p* < 0.001), with both activities being more prevalent in juveniles than in adult males and females. However, grooming (G) and aggression (Xa) were conducted less likely by juveniles compared to adults (Figure 4).

##### 3.3.1.3 Season

**FIGURE 5:**
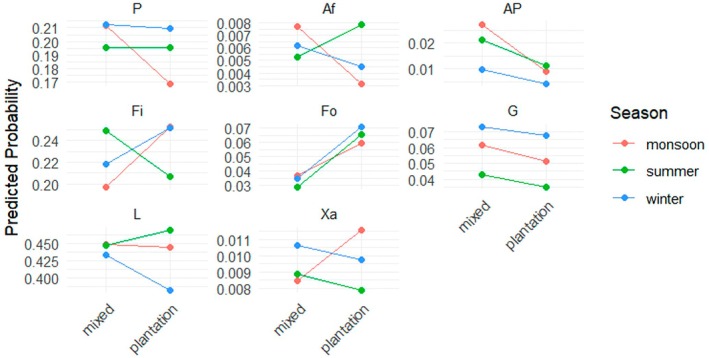
Probabilities predicted for each activity of *Macaca leonina* according to habitat type (mixed or plantation) and season (monsoon, winter, or summer) as estimated from the multinomial regression model (Appendix 8). The *y*‐axis represents the probability predicted by the model of observing a given activity, ranging from 0 to 1. Affiliative behaviors (Af, *p* = 0.043) and feeding (Fi, *p* < 0.001) were significantly influenced by habitat regarding the interaction with season. Af, Affiliative behaviors; AP, Playing; Fi, Feeding; Fo, Foraging; G, Grooming; L, Locomotion; P, Positioning; Xa, Aggression.

There was only one significant effect of season on the activity budget that was independent of the interaction with habitat: playing (AP, *p* = 0.002) was less likely to be observed in winter than during the monsoon or summer. For the interactions between season and habitat, the model revealed that affiliative behaviors (Af, *p* = 0.043) were significantly more likely in summer in the plantation habitat compared to summer in the mixed habitat or monsoon/winter in the plantation. However, feeding (Fi, *p* < 0.001) was significantly less likely to be observed in the plantation in summer compared to summer in the mixed habitat or winter/monsoon in the plantation habitat (Figure 5).

##### 3.3.1.4 Stratum

Regardless of habitat type, affiliative behaviors (Af, *p* < 0.001) occurred less likely at the stratum levels F1 or F2 (both *p* < 0.001) compared to F0. Playing (AP) occurred significantly less likely in F2 compared to F0 (*p* < 0.001). Feeding, foraging, and grooming were significantly less likely to occur at levels F1 (Fi, *p* < 0.001; Fo, *p* < 0.001; G, *p* = 0.012) or F2 (Fi, *p* < 0.001; Fo, *p* < 0.001; G, *p* = 0.026) in the plantation habitat compared to the level F0 in the plantation habitat or F1 and F2 in the mixed habitat. Locomotion was more likely to be observed at level F1 (L, *p* < 0.001) and F2 (L, *p* = 0.007) in the plantation habitat compared to F0 in the plantation habitat or F1 and F2 in the mixed habitat.

### 3.4 Stratum Use

Macaques spent almost half (45.1%) of the daytime in the middle habitat strata between 10 and 25 m above ground (F2), almost one third (29.6%) in the lower stratum up to 10 m above ground (F1), and one quarter (24.9%) of their time on the ground (F0). Macaques rarely (0.5%) used the highest stratum (F3: above 25 m).

#### 3.4.1 Factors Influencing Stratum Use

To explore the effects of habitat, sex–age class, and season on the stratum use of macaques, we fitted the multinomial regression model with the “stratum” as a response variable with F0 (ground) as a reference level, excluding F3 (Appendix 9).

##### 3.4.1.1 Habitat Type

**FIGURE 6:**
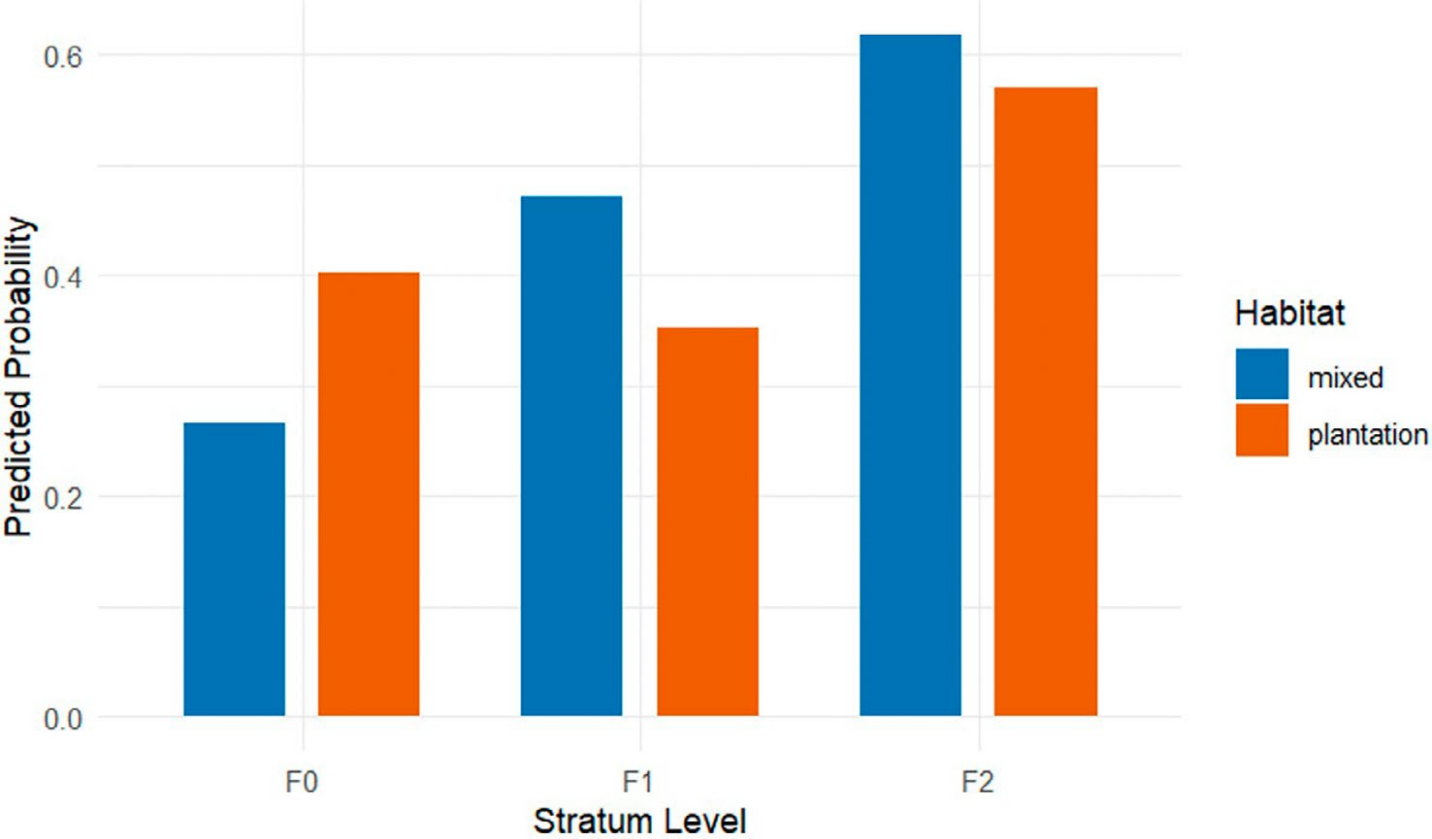
Probabilities predicted of the vertical stratum use of *Macaca leonina* according to habitat type (mixed or plantation) as estimated from the multinomial regression model (Appendix 10). The *y*‐axis represents the probability of observing an individual at the respective stratum level, with F0 being the ground and F2 the highest level included in the model, ranging from 0 to 1. Macaques in the plantation habitat used F0 more frequently, whereas macaques in the mixed habitat used F1 and F2 more often compared to the respective other group.

Macaques in the mixed habitat were more likely to occupy strata F1 and F2 (both *p* < 0.001) than the group in the plantation habitat, who occupied F0 more often (Figure 6). However, there were several significant interactions that influenced the habitat effect.

##### 3.4.1.2 Age and Sex

**FIGURE 7:**
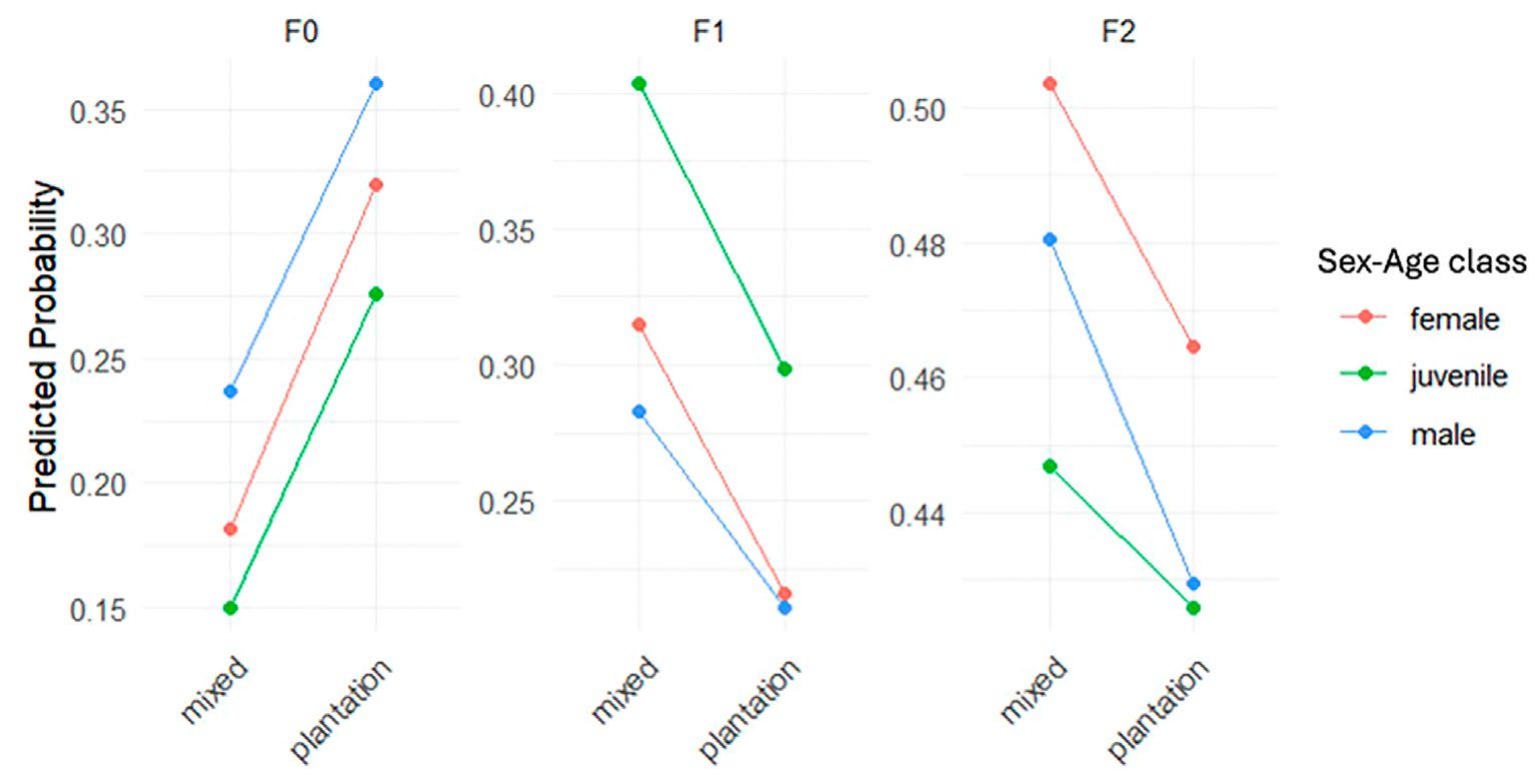
Stratum use of *Macaca leonina* according to habitat type (mixed or plantation) and sex–age class (adult male, adult female or juvenile of both sexes) as estimated from the multinomial regression model (Appendix 10). The *y*‐axis represents the probability predicted by the model of observing a given activity, ranging from 0 to 1. The interaction between habitat and sex–age class shows a trend for females in stratum F1.

The interaction between age and habitat was not significant. Juveniles in both habitats spent significantly more time in stratum F1 (*p* < 0.001) compared to adults of both sexes but did not show significant differences from adults in stratum F2. Adult females were significantly more likely to be observed in stratum F2 compared to adult males regardless of habitat (*p* < 0.001). In stratum F1, the interaction between sex and habitat was almost significant (*p* = 0.052), and adult females showed a higher probability of observation in stratum F1 in the mixed habitat compared to males in the mixed habitat and adult females in the plantation habitat (Figure 7).

##### 3.4.1.3 Season

The interaction between season and habitat significantly influenced the stratum use of macaques. Compared to baseline F0, both in summer and winter, the odds that they occupy stratum F1 (*p* = 0.004 and *p* = 0.002, respectively) or stratum F2 (*p* = 0.030 and *p* < 0.001, respectively) in the plantation habitat increased significantly compared to monsoon and the mixed habitat reference level.

### 3.5 Dietary Diversity

Plant‐based dietary diversity did not differ between habitats. Shannon–Wiener indices (*H*′) calculated for plant taxa were 2.178 in the mixed habitat (*S* = 35) and 2.395 in the plantation (*S* = 36), and Hutcheson's *t*‐test indicated no significant difference (*t* = −0.61, df = 1567, *p* = 0.543). Evenness was moderately high at both sites but slightly greater in the plantation (*J* = 0.675) than in the mixed habitat (*J* = 0.608), indicating a more equitable distribution of feeding effort among plant species in the plantation. Invertebrate consumption in the plantation was notably higher (16.8% of all “feeding” activities) compared to the mixed habitat (7.2%).

## 4 Discussion

This study revealed clear differences in activity budgets and stratum use between two groups of northern pig‐tailed macaques ( *Macaca leonina* ) occupying different habitat types in and around the Satchari National Park: a mixed forest plantation mosaic and a polycrop plantation without natural forest access. These differences appear to be shaped primarily by habitat structure and resulting food availability but are further modulated by sex–age classes and seasonal variation.

### 4.1 Factors Influencing Macaques Activity Budgets

Our regression models confirmed habitat as a significant predictor of feeding and foraging behavior, independent of sex and age. Overall, the plantation habitat (PH) group spent significantly more time feeding and foraging than the mixed habitat (MH) group, consistent with observations in ecologically similar southern pig‐tailed macaques ( *M. nemestrina* ) in oil palm‐dominated habitats in Malaysia (Ruppert et al. 2018). *Macaca leonina* increases foraging activities when fruits are more abundant (Gazagne et al. 2020). Clumped, predictable resources shorten locomotion search time between food patches (Altmann and Muruthi 1988), possibly explaining why PH macaques fed more in the plantation. Foraging time budgets in PH macaques were also significantly higher than in MH, likely stemming from prolonged effort to find invertebrates (see Dietary Diversity below). Plantation diets—limited to mostly cultivated and non‐native plant species—may offer lower nutritional diversity compared to native forest species, potentially requiring higher food intake (Alpízar et al. 2020) and supplementation by invertebrates to meet energy needs. Overall, the combination of higher locomotion and lower feeding and foraging time budgets—but similar positioning time—of our study macaques compared with *M. leonina* in Lawachara National Park, Bangladesh, a large semi‐evergreen forest (Shalauddin et al. 2020: feeding: 30.9%, foraging: 9.9%, locomotion: 34.8%, resting/positioning: 20.4%), suggests activity budget adjustments to lower overall food availability and diversity at our site.

Locomotion dominated the daily activity budgets of both groups, comprising almost half of all observed behaviors (Appendix 6). This aligns with other studies on *M. leonina* in Bangladesh (Shalauddin et al. 2020) and India (Singh et al. 2022), although Choudhury (2008) found resting (positioning) as predominant in Assam forests. Higher locomotion generally reflects lower food availability or patchily distributed food resources, as primates must travel farther to meet their energetic needs. These patterns have been observed in *M. leonina* in Thailand, where longer travel distances corresponded to seasonal fruit scarcity (Albert et al. 2013; Gazagne et al. 2020), and in other Cercopithecidae primates inhabiting fragmented forests, such as colobus monkeys and langurs (Wong and Sicotte 2007; Mekonnen et al. 2018). Being highly frugivorous (Albert et al. 2013; Shalauddin et al. 2020), *M. leonina* searches for ripe fruits that are usually unevenly distributed in natural habitats. A slightly higher locomotion time budget (*p* = 0.053) in the MH group supports this, possibly reflecting habitat heterogeneity and wider spatial distribution of forest food sources compared to the plantation habitat. In contrast, reliable resources in plantations, particularly year‐round fruiting species like oil palm, may reduce the need for prolonged locomotion to travel between food patches.

Positioning comprised about one fifth of the activity budget in our study macaques (Appendix 6), which is similar to *M. leonina* at Lawachara National Park (resting: 20.4%, Shalauddin et al. 2020). In the MH group, higher locomotion was coupled with a slightly higher positioning time budget, likely as recovery from increased energy expenditure from travel (Dunbar 1992). Seasonal positioning peaks in winter, particularly in the PH group, may reflect thermoregulatory adaptations (Thompson and Hermann 2024) and reduced foraging pressure compared to group MH when certain cultivated foods like oil palm remained available in the plantation (Gazagne et al. 2020).

Social behaviors, including grooming, represented less than 10% of the macaques' total activity budget (Appendix 6), but were more common in MH than PH macaques, with playing occurring significantly more often in group MH (*p*_adj < 0.001). During our ad libitum observations, we saw that social behaviors mostly occurred during daytime resting periods when the individuals engaged in playing, mating, grooming, and other affiliative behaviors. Juveniles in both groups engaged more in play and locomotion than adults, consistent with developmental patterns in macaques (Thierry et al. 2000), while adult males in PH showed reduced grooming and play compared to group MH, possibly reflecting greater vigilance requirements of PH males (Caselli et al. 2021; Ruppert et al. 2018). The dense and more complex vegetation structure in the mixed habitat likely offers safer and more comfortable sites for social interactions, while sparser vegetation in the more open plantation habitat may discourage prolonged social bouts due to perceived predation risk (Caselli et al. 2021; Holzner, Balasubramaniam, et al. 2021). Reduced habitat complexity in plantations also constricts macaques in their ability to utilize three‐dimensional space, and individuals are forced to engage in social behaviors more often on the ground than in a more complex habitat, making them potentially more susceptible to threats from the ground (such as feral dogs or humans; Holzner, Balasubramaniam, et al. 2021; Holzner et al. 2024) and therefore warier of engaging at all. However, at our study site, which is seasonally crowded with visitors, no poachers or predators were observed during the study period, and both study groups were well‐adapted to the presence of tourists, local firewood collectors and other people. In Bangladesh, this species has no distinct reproductive season that could potentially influence social behaviors such as mating, grooming, nursing, or playing (M. Feeroz 2003).

Multinomial regression further showed that habitat significantly influenced feeding (*p* < 0.001) and affiliative behaviors (*p* = 0.043) through seasonal interactions. Feeding probabilities in the PH group were higher during monsoon and winter but declined markedly in summer, falling below both other plantation seasons and the mixed habitat. This seasonal depression likely reflects interacting nutritional and thermal constraints. Although oil palms fruit year‐round, the multi‐crop plantation may offer fewer nutritionally complementary plant taxa during summer. In addition, reduced vegetation cover in the plantation increases heat exposure compared to the shaded mixed forest. Elevated thermal stress can suppress feeding‐related activities as individuals adjust activities to mitigate heat load (Dunbar 1992). Thus, lower summer feeding in PH macaques likely reflects combined phenological and thermoregulatory pressures. In contrast, affiliative behaviors (excluding grooming) showed the opposite seasonal trend, increasing in PH during summer when feeding declined. This pattern suggests that low‐cost affiliative interactions (e.g., embrace, touch, friendly mount) may increase when individuals aggregate in shaded microsites or when ecological stress elevates social tension. Grooming, a more time‐ and energy‐intensive behavior, did not show this increase, indicating that energetically more costly social behaviors may remain constrained under heightened foraging (Dunbar 1992) or thermal demands. Together, these results suggest that structural simplifications that alter resource dynamics and microclimate are likely to shape seasonal shifts in feeding intensity and social expression.

### 4.2 Dietary Diversity

Although plant‐based dietary diversity did not significantly differ between both groups, PH macaques exhibited significantly higher probabilities of feeding and foraging, together with a marked increase in invertebrate consumption. These results indicate that behavioral allocation in the plantation habitat was likely shaped less by taxonomic diversity of plant food and more by structural aspects and nutritional constraints. Vegetation assessments revealed pronounced differences in habitat structure. The mixed habitat site consisted of a heterogeneous vegetation mosaic with greater vertical complexity and irregularly distributed food trees, whereas the plantation habitat was structurally simplified and strongly dominated by monocultural oil palm patches, which accounted for 43.4% of relative importance of the site (compared to 8.7% in the mixed habitat). Although oil palms fruit all year‐round, many other plantation species (e.g., *Acacia* sp.: Carron and Aken 1992) exhibit more seasonal reproductive cycles and contribute limited edible resources outside specific phenophases. Consequently, despite comparable species richness of both habitat sites, functional macaque food diversity and seasonal buffering may be markedly reduced in the plantation.

In contrast, the mixed habitat likely benefits from asynchronous fruiting across multiple forest families (e.g., Moraceae, Euphorbiaceae), promoting temporal complementarity and stabilizing food availability (van Schaik et al. 1993). Overall, flowering and fruiting periods of edible native forest plant species in Bangladesh are highly variable, and forest fruits are available year‐round with most fruiting species producing fruit mainly between March and September (Pasha and Uddin 2019).

Despite the clumped distribution of fruit resources in the plantation, PH macaques allocated more time to foraging. A key mechanism underlying this pattern appears to be the pronounced higher consumption of invertebrates in the plantation (16.8%) vs. in the mixed habitat (7.2%). Extractive invertebrate foraging typically involves dispersed searching, substrate manipulation, and longer handling times relative to fruit feeding (Lambert 1998). Increased reliance on invertebrates may contribute to elevating feeding and foraging time budgets, even in habitats where fruit trees are aggregated. This pattern is consistent with compensatory nutritional strategies whereby animals increase effort or diversify trophic intake to meet macronutrient targets when diets are compositionally constrained (Felton et al. 2009). The significantly reduced play and grooming behaviors, particularly among adult males in the PH group, suggest energetic trade‐offs under heightened foraging demands. When food acquisition requires increased extractive effort or dietary supplementation, time allocated to affiliative behaviors may decline. Thus, behavioral allocation appears sensitive to nutritional landscape and structural habitat simplification.

Collectively, these findings indicate that comparable dietary diversity indices can potentially mask divergent energetic environments. Although plant dietary breadth was statistically similar between groups, structural dominance by oil palm and increased reliance on extractive invertebrate foraging in the plantation indicate reduced nutritional buffering and elevated energetic investment in food acquisition in PH macaques. Behavioral variability and trophic shifts may therefore provide more sensitive indicators of habitat quality than diversity metrics alone.

### 4.3 Stratum Use and Spatial Behavior

Overall, both groups spent most of their time in the middle and lower strata and less time on the ground. The use of the highest canopy stratum was rare, but interestingly, it occurred significantly more often in the plantation compared to the mixed habitat. Here, the macaques spent their positioning times in the highest trees (ca. 25 m) that were scattered throughout the plantation site. We observed that stratum use by the macaques varied according to daytime (described in Appendix 10). The PH macaques preferred the highest stratum (F3) of emergent trees as night sleeping sites and were recorded here only early in the morning and late in the afternoon, as well as during daytime resting bouts around noon. In the relatively more open plantation habitat, compared to the denser mixed forest habitat, these few remaining highest trees may offer the best protection against predators (Ruppert et al. 2018). Furthermore, the structurally poor monocultural oil palm plots did not offer a middle stratum section for macaques to rest. Contrarily, the MH group mostly used the middle stratum for night and daytime resting, and they were rarely recorded in the highest stratum. This may be due to higher overall connectivity across the middle stratum layer in the mixed habitat offering visual protection and faster escape routes to avoid predators (Albert et al. 2011) compared to emergent, disconnected forest trees. In the later morning, both groups used the middle stratum level, which is consistent with other studies (Albert et al. 2011; Ruppert et al. 2018). At noon, the PH group preferred the ground, with almost half of all observations made here (Appendix 9), to travel between food patches and forage for food, like *M. nemestrina* in Malaysian oil palm plantations (Ruppert et al. 2018).

While our results indicate a generally arboreal behavior of *M. leonina* , as described in other studies (Choudhury 2010), some researchers consider this species semiterrestrial (Albert et al. 2011) or mainly terrestrial (Boonratana et al. 2022). Indeed, the range of terrestriality in macaques seems to depend on site‐specific environmental influences such as canopy cover and habitat quality (e.g., Choudhury 2008, 2010; Zhou et al. 2014). *Macaca leonina* living in a forest with intact canopy cover in Bherjan, Assam almost never came down to the ground whereas in the nearby Borajan, a site with broken canopy, the macaques frequently used the ground to cross roads and clearings (Choudhury 2010). The PH macaques in our study used the ground significantly more than the MH group, a likely consequence of the broken canopy cover and the less complex vertical structure of plantation trees, which provide little horizontal connectivity. This increased terrestriality in degraded habitats has been documented in northern (Choudhury 2010) and southern (Ruppert et al. 2018) pig‐tailed macaques and in other primates experiencing canopy fragmentation (Mekonnen et al. 2018).

Seasonal patterns in stratum use highlight environmental influences. Both groups used the middle stratum more and the ground less in winter, possibly to reduce exposure to cooler ground temperatures (Hanya et al. 2007). Macaques used the high stratum and ground more in summer, likely to access seasonal food sources and for thermoregulation through increased airflow (Thompson and Hermann 2024). The seasonal stratum effect was more pronounced in the plantation habitat, possibly due to less canopy cover between crop plants and different fruiting patterns than in the mixed habitat. During monsoons, macaques generally avoided the ground, possibly to avoid waterlogged conditions, mirroring patterns seen in other tropical primates (Ding and Zhao 2004).

Age and sex also shaped vertical space use. Juveniles preferred the lower and middle strata, likely due to safety and proximity to social partners, while adult males were more terrestrial, possibly for vigilance and competitive access to ground‐level foods (Ruppert et al. 2018; Holzner, Balasubramaniam, et al. 2021). While we observed individuals of all sex–age classes using the ground, Choudhury (2010) reported that solitary *M. leonina* males were the only individuals using the ground in a forest with intact canopy connectivity. At our site, both study groups crossed forest trails and roads (Figure 2; Appendix 2) to move between feeding areas. During ad libitum observations, we saw that most individuals, especially adult females carrying infants and adult males, crossed roads by using the ground, but juveniles and adult females without infants mostly jumped using connecting trees of the lower and middle canopy strata.

Our models also linked stratum use with specific behaviors. This is in line with Choudhury (2010) who stated that *M. leonina* uses the top story (12–35 m) for feeding and roosting and the lower branches and shrubs in the understory (2–10 m) for feeding, resting, and traveling, while the ground is mainly used for crossing clearings (Choudhury 2010). Positioning and social activities were highly more likely in the middle stratum in both habitats, possibly ensuring visual protection and safety from predators. In the plantation habitat, feeding and foraging were less likely to be observed in the middle stratum than in the mixed habitat, likely reflecting the spatial distribution of food resources. In monocultural patches, fruits occur more uniformly in a certain stratum, while edible items are more widely distributed across all forest strata. As noted above, the PH group used ground more during locomotion compared to MH, reflecting less arboreal connectivity in the plantation habitat. This suggests that the vertical dimension of habitat structure directly constrains behavioral opportunities, a finding consistent with studies on Barbary macaques on agricultural edges (Neves et al. 2023).

### 4.4 Conservation Implications

Our results suggest that endangered northern pig‐tailed macaques in degraded habitats in Bangladesh can exhibit substantial behavioral variability enabling persistence across anthropogenically‐modified landscapes. Both study groups ranged near each other within a human‐modified forest‐agriculture mosaic. The PH macaques adjusted their time budgets and stratum use to exploit predictable but nutritionally limited plantation resources within this simplified environment, whereas MH macaques maintained more diverse foraging and movement patterns consistent with structurally heterogeneous habitats.

The greater vertical and floristic complexity of forests provides opportunities for more arboreal movement, diversified foraging and feeding, and enhanced social interactions, highlighting the importance of three‐dimensional habitat features for sustaining natural behavioral repertoire (Arroyo‐Rodríguez and Dias 2010). In simplified plantation systems, reduced canopy connectivity and limited arboreal food diversity promote increased terrestriality and altered social activity, suggesting that structural impoverishment may impose ecological and social costs despite apparent behavioral adjustment (Estrada et al. 1999). Reliance on cultivated resources may entail further hidden costs. Diets dominated by oil palm and other planted crops may not meet all nutritional needs and plantation foraging animals may be exposed to agricultural pesticides and other hazards, potentially affecting their health and reproduction in the long term (Holzner et al. 2024). Furthermore, increased terrestriality of macaques in anthropogenic habitats can increase risks from human disturbance (such as hunting, retaliatory killings, and pet trade), road kills during road crossings, and disease risk from domestic animals (Al‐Razi et al. 2019). Greater terrestriality can also alter the response to sympatric competitors that shape avoidance/coexistence patterns in niche separation, as seen in *M. leonina* that shares habitat with three sympatric macaque species in Xishuangbanna, China (He et al. 2024). The reduced occurrence of social behaviors in the PH group further raises questions about potential impacts on social cohesion and group stability in the long term that could ultimately impact genetic diversity, fitness, and survival of small and fragmented meta‐populations (Holzner, Balasubramaniam, et al. 2021).

Given that all macaques at the study area occupy habitat sites with strong anthropogenic influence, conservation strategies should focus not only on limiting disturbance but, critically, on enhancing habitat structural complexity and resource diversity in degraded patches. Priorities should include restoring canopy connectivity between primate feeding sites, retaining tall trees and midstory vegetation, and promoting vertical stratification within plantation matrices. Integrating native primate food tree species into agricultural landscapes could diversify food resources and reduce reliance on nutritionally restricted crops. Existing community‐based forest regeneration programs at Satchari (BFD 2016) offer valuable opportunities to improve habitat quality by increasing structural heterogeneity and food availability for *M. leonina* and sympatric primates, ultimately improving the coexistence of humans and arboreal wildlife in the area. Strengthening such programs is particularly important in light of ongoing primate declines in Bangladesh, including the recent national extinction of the highly ecologically flexible long‐tailed macaque ( *M. fascicularis* ) (Hansen et al. 2022).

Finally, our findings underscore the importance of considering vertical habitat structure, in addition to horizontal space, when assessing primate habitat quality and behavioral ecology. Stratum use patterns provide valuable insight into how primates and other arboreal and semi‐arboreal species interact with three‐dimensional environments and adjust to habitat simplification and anthropogenic constraints.

## 5 Study Limitations

Although this is the first comprehensive, long‐term behavioral study of the endangered *M. leonina* in Bangladesh, several limitations should be considered when interpreting the results. First, the absence of individual identification precluded focal animal sampling and prevented control for repeated measures, potentially inflating degrees of freedom and warranting cautious interpretation of statistical inferences. Similarly, the lack of identified sex–age classes during group counts did not allow us to test for effects of group composition (i.e., sex ratios, adult‐nonadult ratios) although these factors may influence behaviors. Second, year‐round monitoring of wild primates in complex terrain is logistically demanding and not all three groups present at the site could be included. The exclusion of one group limits the representativeness of the findings, reduces statistical power, and may constrain the generalizability of habitat‐related patterns to the broader population at the site. Third, forested areas within the mixed habitat were partially inaccessible due to dense vegetation and challenging topography, resulting in fewer vegetation plots compared to the plantation habitat. In addition, repeated phenological assessments of flowering and fruiting within plots across multiple months were not feasible due to logistical constraints, including limited manpower and time. The absence of comprehensive, long‐term phenological data at both sites restricts our ability to directly link seasonal variation in resource availability to observed patterns of feeding and foraging behavior. Consequently, inferences regarding ecological drivers of dietary shifts and activity budgets remain indirect and should be interpreted with caution. Finally, the low frequency of observations in the upper canopy may reflect sampling bias, as individuals in higher strata are less detectable during group scans than those in lower strata (Ruppert et al. 2018). Despite the use of binoculars, dense vegetation can obstruct visibility from the ground, a limitation common to observational studies of arboreal mammals.

Despite these limitations, comprehensive long‐term behavioral observations of primates remain the gold standard for understanding their potential for behavioral plasticity and specific spatial and temporal adaptations in changing environments. For example, while the use of terrestrial camera traps has been proven a highly feasible tool to study diverse aspects of faunal ecology and behavior across the world (e.g., Wearn and Glover‐Kapfer 2019), for (semi‐) arboreal and behaviorally flexible macaques, we caution that their complex space use patterns, shaped by a variety of environmental and biotic factors, as shown in this study, should always be considered when making conclusions about their behavioral adaptations to anthropogenic habitats that inform research and conservation strategy planning (Holzner et al. 2025).

## 6 Conclusions

Northern pig‐tailed macaques at Satchari exhibit distinct activity budgets and stratum use patterns shaped by habitat type, with additional modulation by age, sex, and season. The plantation group fed and foraged significantly more, consumed higher proportions of invertebrates, and used the ground more frequently, whereas the mixed‐habitat group played more and used the middle canopy to a greater extent. Plant dietary diversity did not differ significantly between both groups despite strong structural dominance of oil palm in the plantation. However, elevated invertebrate consumption and seasonal shifts in feeding probabilities in the plantation group indicate dietary flexibility and compensatory trophic adjustment in response to habitat simplification and seasonal constraints. These patterns highlight the behavioral and dietary plasticity of *M. leonina* , suggesting an ability to adjust both activity allocation and diet composition in human‐modified landscapes. At the same time, shifts in social behaviors and increased reliance on extractive foraging point to potential energetic trade‐offs associated with long‐term use of structurally simplified habitats. Continued long‐term monitoring integrating behavioral, nutritional, and phenological data will be essential to assess whether these adjustments represent sustainable adaptive strategies for this endangered primate in Bangladesh and across its range.

## Author Contributions

**Habibon Naher:** conceptualization (equal), data curation (equal), formal analysis (supporting), funding acquisition (lead), investigation (lead), methodology (supporting), project administration (equal), resources (lead), supervision (equal), validation (supporting), writing – original draft (equal), writing – review and editing (supporting). **Tania Akhter:** data curation (equal), formal analysis (supporting), investigation (equal), writing – review and editing (supporting). **Asad Ullah:** data curation (equal), investigation (equal), writing – review and editing (supporting). **Md. Shahriar Siam:** data curation (supporting), investigation (equal), writing – review and editing (supporting). **Shawkat Imam Khan:** data curation (supporting), investigation (equal), writing – review and editing (supporting). **Muhammad Saiful Islam:** funding acquisition (supporting), project administration (equal), writing – review and editing (supporting). **Miriam Simon:** formal analysis (lead), methodology (supporting), software (lead), validation (equal), visualization (equal), writing – original draft (supporting), writing – review and editing (supporting). **Nadine Ruppert:** conceptualization (equal), formal analysis (supporting), investigation (equal), methodology (lead), supervision (equal), validation (equal), visualization (equal), writing – original draft (equal), writing – review and editing (lead).

## Funding

This work was supported by the Bangladesh Bureau of Educational Information & Statistics (BANBEIS) Grant for Advanced Research in Education (GARE), Ministry of Education, Government of the Peoples' Republic of Bangladesh.

## Disclosure

Statement of Inclusion: Our study brings together authors from several regions, including the lead scientists based in the study country itself. All contributors were involved from the early stages of research and study design, ensuring that our diverse perspectives were incorporated from the outset. Where relevant, we cited literature by regional scientists and made efforts to include important work published in the local literature.

## Conflicts of Interest

The authors declare no conflicts of interest.

## Data Availability

The data are available to view at https://tinyurl.com/Macacaleonina.

## Appendix 1 Climate

Weather data recorded at the study site (August 2020 through July 2021). Units: temperature (°C), relative humidity (%) and rainfall (mm), MHT: mean highest temperature (°C), MLT: mean lowest temperature (°C), MT: mean temperature (°C), MHH: mean highest humidity (%), MLH: mean lowest humidity (%), MH: mean humidity (%), MHR: mean highest rainfall (mm), MLR: mean lowest rainfall (mm), MR: mean rainfall (mm). Seasons: winter (November to February), summer (March to June), and monsoon (July to October).

## Appendix 2 Groups' Ranging Areas

Daily travel paths of the two study groups of *Macaca leonina* at Satchari National Park, Raghunandan Hill Reserve Forest (24°07′36″ N, 91°27′26″ E; August 2020–July 2021, see Figure 1). Mixed habitat group (MH): Red line; Plantation habitat group (PH): Yellow line. Image created with Google Earth, 2021. The national highway N204 bisects the study site and the home range of group MH.

## Appendix 3 Sex–Age Classes

Defining the individuals based on their morphological characteristics.

**Table:**

Individuals	Characteristics
Adult Male (AM)	Fully grown with adult morphology (prominent testes).
Adult Female (AF)	Fully grown, reproductive female (anogenital swelling and/or elongated nipples).
Juvenile (JUV)	Small non‐reproductive individuals that were weaned but still ranged in frequent proximity to a female.
Infant	Immature, dependent individuals frequently clinging to the mother during movement and resting (not sampled).

## Appendix 4 Feeding Frequencies

Feeding frequencies of food items collected during group scans (activity “feeding”).

**Table:**

Food items	MH (%)	PH (%)		MH (%)	PH (%)
Fruits and flowers					
*Acacia auriculiformis*	0.8	5.3	*Lannea coromandelica*	0.1	0
*Acacia concinna*	0.1	0.0	*Macaranga denticulata*	0	0.1
*Acacia mangium*	0.0	3.2	*Mallotus albus*	0	0.5
*Albizia falcataria*	1.9	3.8	*Microcos paniculata*	6.9	2.5
*Ampelocissus barbata*	0.5	0.1	*Mimosa* sp.	0.4	2.2
*Antidesma ghaesembilla*	0.4	0.2	*Neolamarckia cadamba*	5.4	1.9
*Aporosa octandra*	1.1	0.9	*Schumannianthus dichotomus*	0.9	0.3
*Artocarpus chama*	5.3	6.5	*Streblus asper*	0.1	0.3
*Artocarpus lacucha*	0.8	0	*Swietenia mahagoni*	0	0.4
*Balakata baccata*	1.2	0	*Syzygium cumini*	1.0	1.4
*Bambusa* sp.	12.0	3.8	*Tectona grandis*	0.1	0
*Bauhinia variegata*	1.0	0.4	*Terminalia catappa*	0.1	0.1
*Bombax ceiba*	0.1	0	*Vitex peduncularis*	0.1	0
*Callicarpa* sp.	0.1	0	*Xylia kerrii*	0	0.1
*Carica* sp.	0	0.1	Unidentified plants	1.9	0.4
*Caryota urens*	0.3	0	Invertebrates	7.3	16.1
*Citrus grandis*	0	0.1	Others (bark, leaves, fungi, grass)	3.6	5.1
*Citrus limon*	0.7	0	Total (%)	100.0	100.0
*Coffea arabica*	0.1	0			
*Combretum* sp.	0	0.8			
*Dillenia pentagyna*	0.1	0.2			
*Elaeis guineensis*	38.0	32.1			
*Eucalyptus globulus*	0	0.3			
*Ficus ampelas*	0.0	0.8			
*Ficus auriculata*	0.3	1.9			
*Ficus benghalensis*	0	0.1			
*Ficus elastica*	0	0.6			
*Ficus heterophylla*	0	1.5			
*Ficus hispida*	0	2.2			
*Ficus racemosa*	0.5	3.4			
*Ficus religiosa*	0.1	0.2			
*Ficus tinctoria*	4.0	0			
*Ficus virens*	0.5	0			
*Flacourtia indica*	0.5	0			
*Garcinia cowa*	0.6	0			
*Ilex umbellulata*	1.3	0.1			

## Appendix 5 Group Sizes

Monthly maximum group sizes (August 2020–July 2021). The group sizes varied during the study period due to fission‐fusion group dynamics (M. M. Feeroz 1999). Ahmed and Naher (2021) report a sex ratio of 1:1 and an adult to nonadult ratio of 1:1.2 from three *Macaca leonina* groups at this site from 2018 to 2019.

**Table:**

Months	MH group (number of individuals)	PH group (number of individuals)
August 2020	60	85
September 2020	69	37
October 2020	75	96
November 2020	150	72
December 2020	138	81
January 2021	107	93
February 2021	65	75
March 2021	67	125
April 2021	138	87
May 2021	72	36
June 2021	46	56
July 2021	92	59
Mean	89.9	75.2
Std	35.0	25.5

## Appendix 6 Overall Comparison of All Activities

Overall comparison of all activities between two social groups of *Macaca leonina* at Satchari National Park (August 2020–July 2021). MH: mixed habitat group, PH: plantation habitat group (*N* = 14,591).

**Table:**

Type of activity		Overall		Group MH		Group PH		Overall Pearson's chi squared test, *χ* ^2^ = 124.75, df = 8, *p* < 0.001
		Frequency (*n*)	%	Frequency (*n*)	%	Frequency (*n*)	%	
Feeding		3321	22.8	1681	21.5	1640	24.2	*χ* ^2^ = 10.952, *p*_adj = 0.003
Foraging		690	4.7	251	3.2	439	6.5	*χ* ^2^ = 72.118, *p*_adj < 0.001
Locomotion		6304	43.2	3447	44.2	2857	42.1	*χ* ^2^ = 4.952, *p*_adj = 0.053
Positioning		2886	19.8	1613	20.7	1273	18.8	*χ* ^2^ = 4.754, *p*_adj = 0.053
Social behaviors	Grooming	806	5.5	453	5.8	353	5.2	*χ* ^2^ = 3.516, *p*_adj = 0.091
	Playing	231	1.6	162	2.0	69	1.0	*χ* ^2^ = 34.582, *p*_adj < 0.001
	Mating	126	0.8	70	0.9	56	0.8	*χ* ^2^ = 0.123, *p*_adj = 0.817
	Aggressive	142	1.0	76	1.0	66	0.9	*χ* ^2^ = 0.020, *p*_adj = 0.889
	Affiliative	85	0.6	51	0.7	34	0.5	*χ* ^2^ = 1.272, *p*_adj = 0.334
Total (*N*)		14,591	100	7804	100	6787	100	

## Appendix 7 Seasonal Activity Budgets

Comparison of overall percentages of diurnal activities of two groups of *Macaca leonina* according to seasons in 2020–21 (MH = mixed habitat group, PH = plantation habitat group; N_MH_ = 7284, N_PH_ = 6787; monsoon *χ* ^2^ = 73.7, df = 8, *p*_adj < 0.001; summer *χ* ^2^ = 41.3, df = 8, *p*_adj < 0.001; winter *χ* ^2^ = 59.4, df = 8, *p*_adj < 0.001).

**Table:**

Activities		Summer			Monsoon			Winter		
		Overall (%)	MH (%)	PH (%)	Overall (%)	MH (%)	PH (%)	Overall (%)	MH (%)	PH (%)
Feeding		22.5	25.0	21.8	22.7	19.4	31.4	23.3	21.7	25.0
Foraging		4.6	2.9	4.5	4.7	3.6	0.7	5.2	3.4	7.0
Locomotion		46.3	44.2	48.7	43.6	44.6	42.5	40.5	43.2	37.7
Positioning		19.0	19.4	18.6	19.0	21.0	17.0	20.8	21.0	20.5
Social activities	Grooming	3.8	4.3	3.3	5.5	6.0	5.0	6.9	7.2	6.6
	Playing	1.7	2.1	1.3	2.1	2.7	1.3	0.8	1.0	0.6
	Mating	0.5	0.7	0.3	0.9	1.1	0.7	1.0	0.8	1.2
	Aggressive	1.0	0.9	0.8	0.9	0.8	1.0	1.0	1.1	1.0
	Affiliative	0.6	0.5	0.7	0.6	0.8	0.4	0.5	0.6	0.4
	Total (%)	7.6	8.5	6.4	10.0	11.4	8.4	10.2	10.7	9.8
Total		100	100	100	100	100	100	100	100	100
*n*		3575	1913	1662	5039	2616	2423	5457	2755	2702

## Appendix 8 Multinomial Logistic Regression Model

Results on activity budgets of *Macaca leonina* and the influence of habitat, sex, age, seasons and stratum and the respective interactions. Shown are the estimates, standard error, odds ratio, lowest and highest confidence interval and the *p*‐values for each predictor per response level compared to the baseline P‐positioning (Af, Affiliative behavior; AP, Playing; Fi, Feeding; Fo, Foraging; G, Grooming; L, Locomotion; Xa, Aggression).

**Table:**

Response level	Term	Estimate	Std. error	OR	CI_Low	CI_High	*p*
Af	(Intercept)	−1.250	0.341	0.287	0.147	0.559	0.000
	Habitat:Plantation	−1.602	0.598	0.201	0.062	0.650	**0.007**
	Sex:Male	−1.153	0.377	0.316	0.151	0.661	**0.002**
	Season:Summer	−0.328	0.395	0.720	0.332	1.562	0.406
	Season:Winter	−0.003	0.340	0.997	0.512	1.941	0.993
	Age:Juvenile	−0.394	0.189	0.675	0.466	0.977	**0.037**
	Stratum:F1	−1.323	0.358	0.266	0.132	0.537	**0.000**
	Stratum:F2	−2.532	0.394	0.080	0.037	0.172	**0.000**
	Habitat:Plantation*Age:Juvenile	−0.023	0.707	0.977	0.244	3.908	0.974
	Habitat:Plantation*Sex:Male	1.546	0.548	4.691	1.602	13.735	**0.005**
	Habitat:Plantation*Season:Summer	1.189	0.614	3.283	0.985	10.941	**0.053**
	Habitat:Plantation*Season:Winter	0.524	0.595	1.689	0.526	5.421	0.378
	Habitat:Plantation*Stratum:F1	−0.065	0.604	0.937	0.287	3.064	0.915
	Habitat:Plantation*Stratum:F2	0.388	0.572	1.473	0.480	4.522	0.498
AP	(Intercept)	−2.617	0.379	0.073	0.035	0.153	0.000
	Habitat:Plantation	−0.413	0.594	0.662	0.206	2.121	**0.000**
	Sex:Male	−0.903	0.458	0.405	0.165	0.994	**0.049**
	Season:Summer	−0.090	0.213	0.914	0.603	1.387	0.674
	Season:Winter	−0.741	0.240	0.477	0.298	0.763	**0.002**
	Age:Juvenile	1.039	0.138	2.828	2.157	3.707	**0.000**
	Stratum:F1	0.124	0.310	1.132	0.617	2.078	0.689
	Stratum:F2	−1.522	0.341	0.218	0.112	0.426	**0.000**
	Habitat:Plantation*Age:Juvenile	−0.645	0.455	0.525	0.215	1.280	0.157
	Habitat:Plantation*Sex:Male	−19.756	0.000	0.000	0.000	0.000	**0.000**
	Habitat:Plantation*Season:Summer	0.257	0.391	1.293	0.601	2.782	0.511
	Habitat:Plantation*Season:Winter	0.003	0.456	1.003	0.410	2.454	0.994
	Habitat:Plantation*Stratum:F1	0.019	0.501	1.019	0.382	2.724	0.969
	Habitat:Plantation*Stratum:F2	0.493	0.532	1.638	0.577	4.647	0.354
Fi	(Intercept)	0.025	0.149	1.026	0.767	1.373	0.864
	Habitat:Plantation	1.202	0.188	3.326	2.301	4.807	**0.000**
	Sex:Male	−0.211	0.086	0.810	0.684	0.959	**0.015**
	Season:Summer	0.312	0.093	1.366	1.140	1.638	**0.001**
	Season:Winter	0.098	0.086	1.103	0.932	1.304	0.253
	Age:Juvenile	−0.011	0.045	0.990	0.906	1.081	0.815
	Stratum:F1	−0.003	0.148	0.997	0.746	1.332	0.983
	Stratum:F2	−0.024	0.139	0.976	0.743	1.282	0.861
	Habitat:Plantation*Age:Juvenile	0.040	0.130	1.041	0.807	1.344	0.757
	Habitat:Plantation*Sex:Male	−0.065	0.126	0.937	0.733	1.199	0.606
	Habitat:Plantation*Season:Summer	−0.608	0.137	0.544	0.416	0.712	**0.000**
	Habitat:Plantation*Season:Winter	−0.140	0.124	0.869	0.681	1.109	0.260
	Habitat:Plantation*Stratum:F1	−0.747	0.192	0.474	0.325	0.690	**0.000**
	Habitat:Plantation*Stratum:F2	−1.114	0.174	0.328	0.233	0.461	**0.000**
Fo	(Intercept)	−1.144	0.239	0.319	0.199	0.509	0.000
	Habitat:Plantation	1.596	0.282	4.933	2.838	8.574	**0.000**
	Sex:Male	−0.364	0.174	0.695	0.494	0.977	**0.036**
	Season:Summer	−0.161	0.183	0.851	0.595	1.217	0.377
	Season:Winter	−0.011	0.159	0.989	0.725	1.351	0.947
	Age:Juvenile	0.102	0.082	1.107	0.943	1.300	0.213
	Stratum:F1	−0.465	0.238	0.628	0.394	1.001	**0.051**
	Stratum:F2	−0.736	0.224	0.479	0.309	0.743	**0.001**
	Habitat:Plantation*Age:Juvenile	−0.275	0.220	0.759	0.493	1.169	0.211
	Habitat:Plantation*Sex:Male	0.246	0.221	1.279	0.830	1.971	0.265
	Habitat:Plantation*Season:Summer	0.223	0.238	1.250	0.784	1.992	0.349
	Habitat:Plantation*Season:Winter	0.394	0.211	1.483	0.981	2.242	0.061
	Habitat:Plantation*Stratum:F1	−2.012	0.311	0.134	0.073	0.246	**0.000**
	Habitat:Plantation*Stratum:F2	−1.904	0.265	0.149	0.089	0.251	**0.000**
G	(Intercept)	−1.682	0.284	0.186	0.107	0.325	0.000
	Habitat:Plantation	0.814	0.345	2.258	1.148	4.442	**0.018**
	Sex:Male	−0.396	0.127	0.673	0.524	0.864	**0.002**
	Season:Summer	−0.279	0.152	0.756	0.562	1.018	0.066
	Season:Winter	0.135	0.124	1.144	0.898	1.458	0.275
	Age:Juvenile	−0.203	0.068	0.816	0.714	0.933	**0.003**
	Stratum:F1	0.673	0.287	1.959	1.116	3.439	**0.019**
	Stratum:F2	0.772	0.276	2.164	1.260	3.715	**0.005**
	Habitat:Plantation*Age:Juvenile	0.294	0.196	1.342	0.915	1.969	0.133
	Habitat:Plantation*Sex:Male	−0.448	0.200	0.639	0.431	0.946	**0.025**
	Habitat:Plantation*Season:Summer	−0.238	0.232	0.788	0.501	1.241	0.304
	Habitat:Plantation*Season:Winter	−0.102	0.186	0.903	0.626	1.301	0.583
	Habitat:Plantation*Stratum:F1	−0.917	0.363	0.400	0.196	0.815	**0.012**
	Habitat:Plantation*Stratum:F2	−0.749	0.331	0.473	0.247	0.905	**0.024**
L	(Intercept)	2.066	0.118	7.891	6.262	9.943	0.000
	Habitat:Plantation	−0.237	0.160	0.789	0.577	1.079	0.138
	Sex:Male	0.020	0.080	1.020	0.873	1.193	0.799
	Season:Summer	0.069	0.085	1.071	0.907	1.265	0.418
	Season:Winter	0.140	0.077	1.151	0.990	1.338	0.068
	Age:Juvenile	0.199	0.041	1.220	1.125	1.322	**0.000**
	Stratum:F1	−1.289	0.115	0.275	0.220	0.345	**0.000**
	Stratum:F2	−2.256	0.110	0.105	0.085	0.130	**0.000**
	Habitat:Plantation*Age:Juvenile	−0.097	0.120	0.907	0.716	1.149	0.418
	Habitat:Plantation*Sex:Male	−0.087	0.117	0.916	0.729	1.152	0.454
	Habitat:Plantation*Season:Summer	−0.089	0.125	0.915	0.716	1.168	0.475
	Habitat:Plantation*Season:Winter	−0.168	0.114	0.846	0.676	1.058	0.143
	Habitat:Plantation*Stratum:F1	0.605	0.158	1.831	1.343	2.498	**0.000**
	Habitat:Plantation*Stratum:F2	0.389	0.146	1.475	1.107	1.966	**0.008**
Xa	(Intercept)	−2.682	0.406	0.068	0.031	0.152	0.000
	Habitat:Plantation	0.165	0.559	1.180	0.395	3.528	0.767
	Sex:Male	1.319	0.328	3.738	1.967	7.103	**0.000**
	Season:Summer	0.132	0.331	1.141	0.596	2.184	0.691
	Season:Winter	0.387	0.293	1.473	0.830	2.615	0.186
	Age:Juvenile	−0.349	0.269	0.706	0.417	1.194	0.194
	Stratum:F1	−0.971	0.326	0.379	0.200	0.718	**0.003**
	Stratum:F2	−1.813	0.319	0.163	0.087	0.305	**0.000**
	Habitat:Plantation*Age:Juvenile	1.005	0.676	2.732	0.726	10.274	0.137
	Habitat:Plantation*Sex:Male	−0.205	0.477	0.815	0.320	2.075	0.667
	Habitat:Plantation*Season:Summer	−0.629	0.476	0.533	0.210	1.354	0.186
	Habitat:Plantation*Season:Winter	−0.503	0.414	0.605	0.269	1.361	0.224
	Habitat:Plantation*Stratum:F1	0.395	0.465	1.484	0.596	3.695	0.396
	Habitat:Plantation*Stratum:F2	0.324	0.443	1.383	0.581	3.294	0.464

*Note:* Bold values represent significant *p*‐values (*p* ≤ 0.05).

## Appendix 9 Multinomial Logistic Regression Model

Results on stratum use of *Macaca leonina* and the influence of habitat, sex, age, and seasons and the respective interactions. Shown are the estimates, standard error, odds ratio, lowest and highest confidence interval and the *p*‐values for each predictor per response level compared to the baseline F0 (ground). The response level F3 (>25 m) was omitted from the model due to a lack of sufficient observations.

**Table:**

Response level	Term	Estimate	Std. error	OR	CI_Low	CI_High	*p*
F1	(Intercept)	0.726	0.074	2.066	1.787	2.388	0.000
	Season:Summer	−0.227	0.083	0.797	0.677	0.938	**0.006**
	Season:Winter	−0.345	0.081	0.708	0.604	0.830	**0.000**
	Habitat:Plantation	−1.149	0.103	0.317	0.259	0.388	**0.000**
	Age:Juvenile	0.220	0.043	1.246	1.145	1.356	**0.000**
	Sex:Male	−0.374	0.082	0.688	0.586	0.808	**0.000**
	Season:Summer*Habitat:Plantation	0.337	0.116	1.401	1.117	1.758	**0.004**
	Season:Winter*Habitat:Plantation	0.345	0.113	1.412	1.131	1.763	**0.002**
	Age:Juvenile*Habitat:Plantation	0.015	0.059	1.015	0.904	1.141	0.798
	Sex:Male*Habitat:Plantation	0.226	0.117	1.254	0.998	1.575	0.052
F2	(Intercept)	0.856	0.071	2.354	2.047	2.706	**0.000**
	Season:Summer	−0.047	0.081	0.954	0.814	1.119	0.563
	Season:Winter	0.443	0.075	1.558	1.344	1.806	**0.000**
	Habitat:Plantation	−0.917	0.096	0.400	0.331	0.482	**0.000**
	Age:Juvenile	0.035	0.041	1.036	0.955	1.124	0.394
	Sex:Male	−0.307	0.075	0.735	0.634	0.852	**0.000**
	Season:Summer*Habitat:Plantation	0.240	0.110	1.271	1.024	1.578	**0.030**
	Season:Winter*Habitat:Plantation	0.492	0.101	1.636	1.341	1.996	**0.000**
	Age:Juvenile*Habitat:Plantation	−0.007	0.055	0.993	0.891	1.106	0.894
	Sex:Male*Habitat:Plantation	0.111	0.102	1.118	0.914	1.366	0.277

*Note:* Bold values represent significant *p*‐values (*p* ≤ 0.05).

## Appendix 10 Daytime Comparison of Stratum Use

Daytime comparison of stratum use of *Macaca leonina* in both habitat types (MH: mixed habitat, PH: plantation habitat). F0: ground, F1: low stratum (1–10 m), F2: middle stratum (10–25 m), F3: high stratum (> 25 m).

The stratum use of each individual across daytime was recorded during each group scan. The day period was classified into early morning (after sunrise to 8:00), morning (8:01–11:30), noon (11:31–15:00), and afternoon (15:00–until sleeping site selection). The daytime periods seasonally changed; sunrise times ranged from 4:30 to 6:45 and sunsets ranged from 17:00 to 19:30. The researchers went near the sleeping tree before sunrise and left the study groups after sunset, after the macaques selected their night sleeping site.
